# Effect of acupuncture on monoaminergic neurotransmitters in animal models of vascular dementia: a preclinical systematic review and meta-analysis

**DOI:** 10.3389/fphys.2026.1811438

**Published:** 2026-05-11

**Authors:** Lixiang Gan, Yuqing Fan, Dongmin Liu, Jialing Zheng, Jize Han, Chuyu Deng, Xiaorong Tang, Nenggui Xu, Zhennan Wu

**Affiliations:** 1 South China Research Center for Acupuncture and Moxibustion, Medical College of Acu-Moxi and Rehabilitation, Guangzhou University of Chinese Medicine, Guangzhou, China; 2 Department of Pediatric Neurosurgery, Guangdong Women and Children Hospital, Guangzhou, China

**Keywords:** acupuncture, meta-analysis, monoaminergic neurotransmitters, synaptic plasticity, vascular dementia

## Abstract

**Background:**

Vascular dementia (VaD) is characterized by progressive cognitive impairment associated with chronic cerebral hypoperfusion. Monoaminergic neurotransmitter dysfunction has been implicated in its pathogenesis. Acupuncture has shown neuroprotective potential in experimental models; however, its regulatory effects on monoaminergic systems remain to be systematically clarified. This study aimed to evaluate the effects of acupuncture on monoaminergic neurotransmitters and cognitive function in animal models of VaD through a preclinical systematic review and meta-analysis.

**Methods:**

In accordance with the PRISMA 2020 guidelines, a comprehensive search across eight English and Chinese databases was conducted from inception to October 2025 to identify randomized controlled animal studies investigating acupuncture in VaD models. Primary outcomes focused on the levels of monoaminergic neurotransmitters, including serotonin (5-HT), norepinephrine (NE), and dopamine (DA). Secondary outcomes encompassed acetylcholine (ACh) levels, long-term potentiation (LTP), and behavioral performance. Risk of bias was systematically assessed using the SYRCLE tool, and statistical synthesis was performed using R software (version 4.3.1).

**Results:**

Nine studies involving 386 rodents were included. Meta-analysis demonstrated that acupuncture elevated the levels of 5-HT (SMD = 1.35), NE (SMD = 2.67), and DA (SMD = 1.43). Furthermore, acupuncture treatment was associated with increased ACh levels (SMD = 3.75) and enhanced synaptic plasticity, as evidenced by improved LTP (SMD = 4.75). Behavioral assessments revealed substantial cognitive improvements, indicated by a reduction in escape latency (SMD = −4.66) and an increased number of platform crossings (SMD = 3.00) in the Morris water maze test.

**Conclusion:**

Acupuncture may ameliorate cognitive impairment in VaD by modulating monoaminergic systems and enhancing synaptic plasticity. However, substantial heterogeneity and small sample sizes underscore the exploratory nature of these findings. To avoid overgeneralization of mechanistic pathways, further rigorous studies are essential.

**Systematic Review Registration:**

https://www.crd.york.ac.uk/prospero/, identifier CRD420251179267.

## 1 Introduction

Vascular dementia (VaD) is one of the most common types of dementia caused by cerebrovascular pathological changes, clinically characterized by progressive cognitive impairment and often accompanied by behavioral, emotional, and neurological abnormalities (O’Brien and Thomas, 2015). As the second most common form of senile dementia after Alzheimer’s disease, VaD accounts for approximately 15%–20% of all dementia cases in North America and Europe, and up to 30% in some parts of Asia (Wolters and Ikram, 2019). The current annual cost for the treatment and care of VaD exceeds 200 billion USD, and with the intensifying global aging population, the economic burden imposed by VaD will become even more severe (Smith, 2017). Currently, the pathogenesis of VaD remains not fully elucidated. It is primarily initiated by abnormal cerebral blood perfusion, which triggers a pathological cascade encompassing hypoxia, oxidative stress, inflammatory responses, and microenvironmental dyshomeostasis (Venkat et al., 2015; Durgapal et al., 2025). Notably, this process not only leads to structural damage in the hippocampus and white matter but also triggers an imbalance in the monoaminergic neurotransmitter system, specifically involving serotonin (5-HT), norepinephrine (NE), and dopamine (DA) (Court and Perry, 2003; Guo et al., 2016; Durgapal et al., 2025). This dual “structural-chemical” impairment synergistically compromises neural transmission and circuitry function, ultimately resulting in cognitive and emotional deficits (Fortin et al., 2002; Court and Perry, 2003; Bells et al., 2017). Existing studies suggest that the dyshomeostasis of monoaminergic neurotransmitters may exacerbate learning, memory, and mood regulation disorders by impairing transmission efficiency and hippocampal- prefrontal cortex (PFC) synaptic plasticity (Ruggiero et al., 2021; Aquilani et al., 2023; Durgapal et al., 2025). Therefore, the onset and progression of VaD are considered to be co-driven by multiple mechanisms, including cerebral hypoperfusion and neurotransmitter system dysfunction. This highlights the need for intervention strategies to simultaneously focus on improving vascular function and modulating neurochemical homeostasis.

Currently, the effective treatment of VaD is significantly more challenging than that of Alzheimer’s disease, as there are no specifically licensed medications (O’Brien and Thomas, 2015). In clinical practice, drugs like donepezil and memantine are frequently used off-label (Noufi et al., 2019; Battle et al., 2021). Although offering some therapeutic benefits, their overall efficacy remains limited since they predominantly target single neurotransmitter pathways. Furthermore, their clinical application is often restricted by notable adverse effects, particularly gastrointestinal and cardiovascular disturbances (O’Brien and Thomas, 2015). Given the stark contrast between the urgent need for disease management and the inadequate efficacy of current pharmacotherapies, there is a pressing need to explore multi-target, comprehensive alternative treatments. Acupuncture, a classic non-pharmacological therapy originating from China, has been increasingly recognized for its advantage in such multi-target network modulation (Ye et al., 2017b; Ji et al., 2021; Liu et al., 2025). In recent years, it has been increasingly utilized as a complementary treatment for various neurological disorders, including stroke, Parkinson’s disease, and dementia (Lu et al., 2022). Recent systematic reviews and meta-analyses have demonstrated that acupuncture treatment can effectively improve cognitive function and activities of daily living in patients with VaD, providing preliminary evidence for its clinical value (Su et al., 2021; Chen et al., 2022; Wen et al., 2022). Mechanistically, current research has preliminarily confirmed that acupuncture exerts its ameliorative effects on VaD pathological damage and functional impairment through pathways such as reducing oxidative stress, inhibiting neuroinflammation, resisting neuronal apoptosis, and modulating synaptic plasticity and neurotransmitter systems (Ye et al., 2017b; Li et al., 2022). Among these, although explorations into the specific mechanism of “acupuncture improving VaD function by modulating monoaminergic neurotransmitters” have accumulated some animal experimental data, existing studies are hindered by scattered results, inconsistent acupuncture intervention parameters, and heterogeneous measurement indicators, lacking a systematic synthesis and evaluation.

Building upon this, the present study aims to conduct a systematic review and meta-analysis to systematically integrate the current experimental evidence regarding the effects of acupuncture on monoaminergic neurotransmitter levels and cognitive function in VaD animal models. We seek to clarify the modulatory effects and underlying patterns of acupuncture in this domain, thereby providing a robust scientific reference for the experimental design and mechanistic exploration of future VaD animal studies.

## 2 Method

### 2.1 Study registration

This study was conducted in accordance with the Preferred Reporting Items for Systematic Reviews and Meta-Analyses 2020 (PRISMA 2020) guidelines (Page et al., 2021), and all items of the PRISMA checklist were strictly followed. Additionally, the study was prospectively registered in the International Prospective Register of Systematic Reviews (PROSPERO) on December 17, 2025, with the registration number CRD420251179267.

### 2.2 Search strategy

As of October 28, 2025, a systematic literature search was conducted across eight databases. The search strategy included four Chinese databases (China National Knowledge Infrastructure, China Science and Technology Journal Database, Wanfang Database and China Biology Medicine) and four English databases (PubMed, Embase, Cochrane Library, and Web of Science Database). Publications in both Chinese and English from all countries were considered. The search strategy included a series of keywords, including (1) “Acupuncture”, “Electroacupuncture” (2); “Vascular dementia”, “ post-stroke dementia “; (3) “animal models”, “Rats”, “Mice”. To combine search terms to obtain results meeting inclusion criteria, Boolean operators such as “AND” and “OR” were used. For these keywords, we utilized a combination of controlled vocabulary (e.g., Medical Subject Headings [MeSH]) and free-text words. For example, the search strategy in the PubMed database explicitly combined these elements and logical operators using specific field tags: ((“Acupuncture”[Mesh] OR Acupuncture[Title/Abstract]) AND (“Dementia, Vascular”[Mesh] OR Vascular dementia[Title/Abstract]) AND (“Models, Animal”[Mesh] OR rats[Title/Abstract])). The complete and reproducible search strategies for each of the eight databases have been summarized in **Supplementary Material 1**.

### 2.3 Literature screening

To ensure a rigorous literature screening process, studies were selected based on the following inclusion criteria: (1) Study design type: Only randomized controlled trials utilizing animal models were included. (2) Subjects: Studies must have successfully established animal models of VaD, with no restrictions on the modeling method, animal species, sex, or age. (3) Interventions: The treatment group must have received manual acupuncture (MA), electroacupuncture (EA), or a combination of MA/EA with other therapies. No restrictions were imposed regarding acupoint selection, manipulation techniques, needle insertion angles, duration per session, total treatment course, EA parameters (e.g., frequency and intensity), or the specific forms of combined therapies. (4) Outcome measures: Primary outcome measures included monoaminergic neurotransmitter levels (e.g., 5-HT, NE, and DA). Secondary outcome measures encompassed acetylcholine (ACh) levels, long-term potentiation (LTP) detection, and cognitive behavioral indicators. Specifically, cognitive performance was assessed using the Morris water maze (MWM) test, evaluated via escape latency (MWM-Escape latency) and the number of platform crossings (MWM-Number of platform crossings). Furthermore, the step-down avoidance task was measured by step-down latency (Step-Down latency) and the number of errors (Number of step-down errors). (5) Publication type and language: Only full-length animal experimental studies published in English or Chinese were included. Exclusion criteria were as follows: (1) Animal experiments not using randomized controlled trial design. (2) Studies in which the treatment group did not receive any form of MA or EA. (3) Duplicate publications or studies with redundant data. (4) Studies that did not include monoaminergic neurotransmitters as outcome measures. (5) Literature with incomplete data, conference abstracts only, or editorial commentaries. (6) Studies determined not to meet the aforementioned inclusion criteria upon manual screening.

### 2.4 Data extraction

Upon completion of the literature search, all retrieved records were imported into EndNote X9 software for deduplication. Subsequently, two independent reviewers (Gan, L.X., Fan, Y.Q.) screened the titles and abstracts of the remaining 982 articles, excluding 970 studies that clearly failed to meet the inclusion criteria. The full texts of the remaining articles were then comprehensively assessed for eligibility. Data extraction was performed by two researchers (Deng, C.Y. and Han, J.Z.), who systematically collected the following predetermined items: basic study characteristics (first author, publication year, and sample size); animal model details (species, sex, age, weight, group allocation, and method of establishing the model); intervention protocols for both treatment and control groups (intervention type, selected acupoints, treatment time and duration, and specific acupuncture parameters); and outcome data (specimen, neurochemical and neuroelectrophysiological indicators, and behavioral tests). For numerical data presented exclusively in graphical format, GetData Graph Digitizer software v2.26 was utilized for accurate extraction. Any discrepancies arising during the process were resolved through consultation with a third reviewer (Wu, Z.N.) to reach a consensus.

### 2.5 Risk of bias assessment

The potential risk of bias for each included study was assessed utilizing the Systematic Review Centre for Laboratory Animal Experimentation (SYRCLE) (Hooijmans et al., 2014) risk of bias tool. This tool comprises 10 items addressing six domains of bias (Selection, Performance, Detection, Attrition, Reporting, and Other), including random sequence generation, baseline characteristics, allocation concealment, random housing, blinding of participants and personnel, random outcome assessment, blinding of outcome assessors, incomplete outcome data, selective outcome reporting, and other sources of bias. Each item was categorized as ‘low risk’ if the respective methodology was appropriately conducted and clearly articulated. Conversely, items were deemed ‘high risk’ or ‘unclear risk’ if the methodological procedures were problematic, ambiguous, or inadequately detailed. Two investigators (Liu, D.M. and Zheng, J.L.) independently evaluated these factors, consulting a third investigator (Wu, Z.N.) when necessary to resolve discrepancies.

### 2.6 Data analysis

All statistical syntheses and meta-analyses were conducted using R software (version 4.3.1) with the meta package, while the ggplot2 package was utilized for the visualization of the combined forest plots. For continuous variables, standardized mean differences (SMD) with 95% confidence intervals (CI) were calculated. Heterogeneity across the included studies was statistically assessed using Cochran’s Q test and quantified by the I^2^ statistic. The choice of the meta-analysis model was strictly determined by the level of heterogeneity: a fixed-effects model was utilized when heterogeneity was low to moderate (I^2^ ≤ 50%), whereas a random-effects model was applied when significant heterogeneity was present (I^2^ > 50%). Additionally, forest plots were generated to visually present the individual effect sizes of each included study alongside the overall pooled estimates. To explore heterogeneity and evaluate robustness, predefined subgroup analyses and leave-one-out sensitivity analyses were conducted. Furthermore, funnel plots and Egger’s test were planned to assess potential publication bias for outcomes with ≥ 10 studies, and meta-regression was planned to explore sources of heterogeneity if there were ≥ 10 studies per covariate.

## 3 Results

### 3.1 Literature screening process

**Figure 1:**
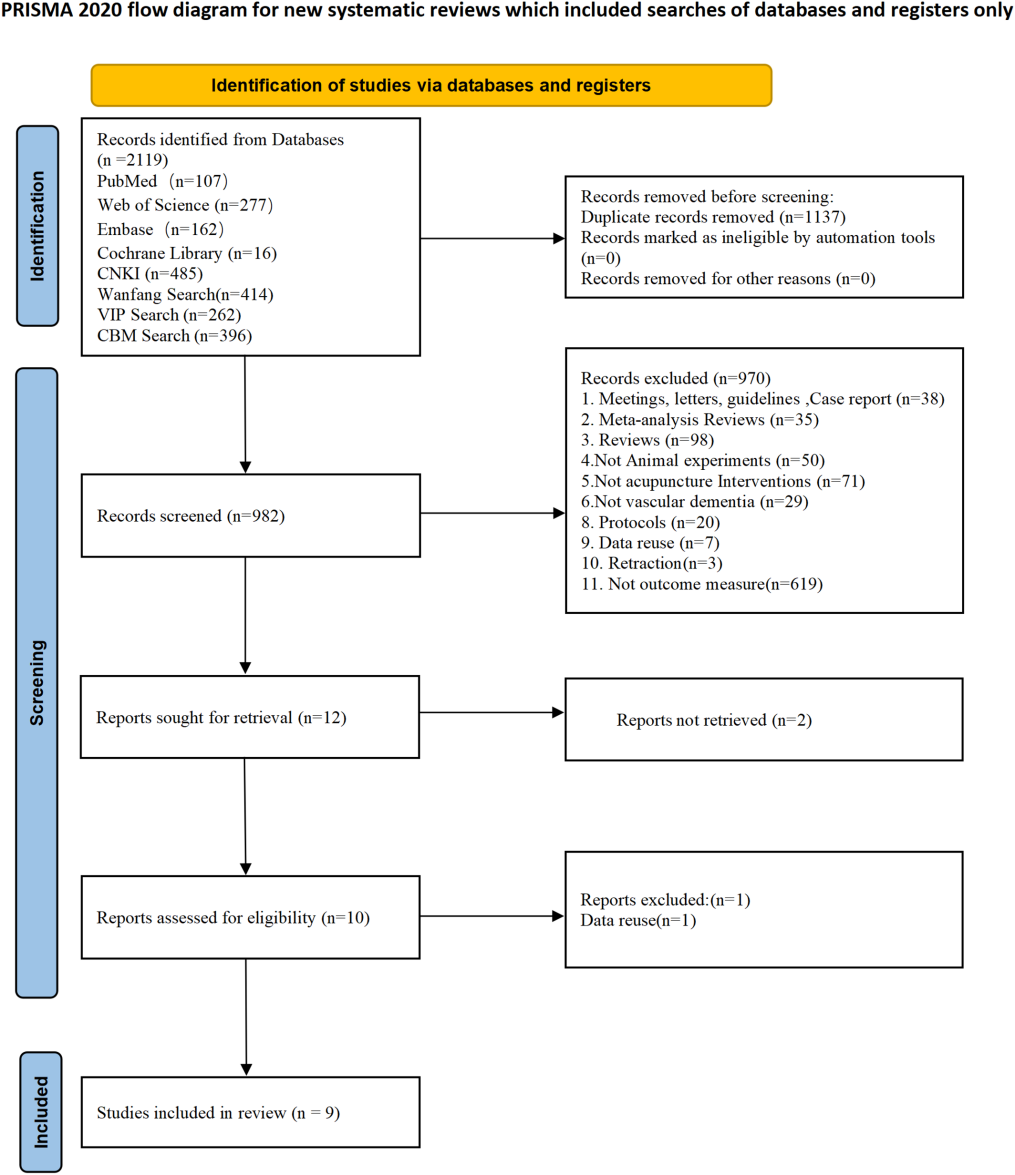
Flow chart of the prisma.

Two independent reviewers (Gan, L.X., Fan, Y.Q.) conducted the literature search, yielding a total of 2,119 records across eight databases based on the search strategy outlined in the protocol. Following the removal of 1,137 duplicate records, the remaining 982 articles underwent initial screening. Preliminary evaluation of titles and abstracts resulted in the exclusion of 970 articles that failed to meet the inclusion criteria, leaving 12 articles for full-text assessment. Two articles were subsequently excluded because their full texts were unobtainable. Upon comprehensive review of the 10 successfully retrieved full texts, one additional article was excluded due to data duplication. Ultimately, nine eligible studies were included in the final meta-analysis. The detailed study selection process and specific reasons for exclusion are delineated in the PRISMA flowchart (**Figure 1**).

### 3.2 Characteristics of included studies

A total of nine studies encompassing 386 rodents were included in this review, with an equal allocation of animals between the treatment and control groups. These studies were published between 1999 and 2025, comprising five articles in Chinese (Lai et al., 1999; Xian, 2004; Ming et al., 2007; Zhang, 2014; Juan et al., 2017) and four in English (Yang et al., 2014; Ye et al., 2017a; Xiao et al., 2018; Wan et al., 2025). Regarding animal models, three studies (Lai et al., 1999; Ming et al., 2007; Juan et al., 2017) utilized Sprague-Dawley (SD) rats, four (Xian, 2004; Yang et al., 2014; Ye et al., 2017a; Xiao et al., 2018) used Wistar rats, one (Wan et al., 2025) employed C57BL/6J mice, and one (Zhang, 2014) selected Kunming mice. Seven studies exclusively utilized male rodents, whereas two studies (Xian, 2004; Yang et al., 2014) incorporated an equal distribution of both sexes. To simulate VaD, five studies (Lai et al., 1999; Yang et al., 2014; Zhang, 2014; Ye et al., 2017a; Xiao et al., 2018) adopted the two-vessel occlusion (2VO) method, and two (Ming et al., 2007; Juan et al., 2017) employed a modified 2VO combined with sodium nitroprusside (SNP) administration. Additionally, one study (Wan et al., 2025) utilized bilateral common carotid artery stenosis (BCAS), and another (Xian, 2004) adopted a thromboembolic multiple infarction dementia model. Notably, none of the included studies reported adverse effects associated with the acupuncture interventions.

Regarding acupoint selection, eight of the nine included studies targeted GV20 (Baihui), while one study (Xian, 2004) omitted this acupoint. Specific combinations included GV20 and GV24 (Shenting) in three studies (Ming et al., 2007; Yang et al., 2014; Wan et al., 2025), and GV20 paired with ST36 (Zusanli) in another three (Zhang, 2014; Ye et al., 2017a; Xiao et al., 2018). However, the selection of supplementary acupoints varied considerably across the remaining literature. In terms of intervention modalities, six studies utilized MA, and three employed EA. In the MA studies, three (Yang et al., 2014; Ye et al., 2017a; Xiao et al., 2018) employed the twirling manipulation, one study (Juan et al., 2017) used even reinforcing-reducing method, one study (Ming et al., 2007) employed the twisting and lifting-thrusting manipulation, and another study did not use any manipulation for reinforcing or reducing. Furthermore, two studies (Juan et al., 2017; Xiao et al., 2018) mentioned acupuncture depth, at 25 mm and 5 mm, respectively. Among the EA studies, two (Lai et al., 1999; Zhang, 2014) applied a dense-sparse wave, while one (Wan et al., 2025) utilized an alternating frequency mode of 2/15 Hz. Overall EA frequencies ranged from 10–12 Hz, 2/15 Hz alternating, to 2–80 Hz. For intensity parameters, one study (Wan et al., 2025) specified 2 mA, and another (Lai et al., 1999) reported a voltage range of 6–10 V; the third EA study (Zhang, 2014) did not disclose specific numerical values but noted that stimulation intensity was meticulously adjusted to induce mild limb trembling without eliciting vocalization or observable struggle.

Data extracted from the nine included studies encompassed five neurochemical and neuroelectrophysiological indicators, alongside two behavioral parameters. For the control group design, five studies employed non-intervention methods, while the remaining four (Xian, 2004; Ye et al., 2017a; Xiao et al., 2018; Wan et al., 2025) utilized sham acupuncture. The principal characteristics of all included studies are summarized in **Table 1**.

**Table 1:** Characteristic of the include studies.

References	Animal models						Sample size	Intervention (treatment group)				Intervention (control group)	Specimen	Neurochemical and neuroelectro- physiological Indicators	Behavioral tests
	Species	Sex	Age	Weight (g)	n=treatment group/ control group	Method of establishing model		Type	Acupoints	Treatment time and duration	Acupuncture parameters				
Dong et al. (2014)	Sprague Dawley rats	Male	2–3 months	200 ± 20	10/10	Modified 2VO combined with SNP	24	MA	GV16,GV20, GV26,CV12, CV16	20 min, once daily for 15 days	The needles were inserted to a depth of approximately 25 mm and were manipulated with even reinforcement and reduction method.	/	Hippocampus	①	a, b
Lai et al. (1999) (Lai et al., 1999)	Sprague Dawley rats	Male	NA	200-250	34/34	2VO	68	EA	GV20,BL23	10 minutes, once every 12 hours starting 24h post-modeling for 3 sessions.	Dense-Sparse Wave, 10–12 Hz, 6-10v	/	Hippocampus,	①②③	/
Tang et al. (2007)	Sprague Dawley rats	Male	4–5 months	250-300	30/30	Modified 2VO combined with SNP	60	MA	BL23,BL18,BL20,GV20,EX-HN1, GV24,GV26,PC6,GB20	30 minutes, once daily for 3 courses (10 days/course) with 1-day intervals	The twisting and lifting interpolation methods are adopted.	/	Cortex	①②③④	c, d,
Wan et al. (2025) (Wan et al., 2025)	C57BL/6J mice	Male	6–8 weeks	NA	21/21	BCAS	42	EA	GV20, GV24	20 min, once every other day for 4 weeks	Alternating frequency of 2/15 Hz and an intensity of 2 mA	Sham acupuncture	Prefrontal Cortex	②	/
Xian (2021)	Wistar rats	half male and half female	10 months	300 ± 40	10/10	Thromboembolic Multiple Infarction Dementia Model	20	MA	CV17,CV12, CV6,SP10, ST36	Once daily for 7 days	All acupoints received 30 seconds of reinforcing method, except SP10 which received reducing method.	Sham acupuncture	Hippocampus	①②③④	a, b
Xiao et al. (2018) (Xiao et al., 2018)	Wistar rats	Male	7–8 weeks	270-300	20/20	2VO	40	MA	GV20, ST36	10min, once daily for 2 weeks, 1 day of rest after six treatments	Needling of selected acupoints to a depth of 5 mm	Sham acupuncture	Hippocampus	②⑤	/
Yang et al. (2014) (Yang et al., 2014)	Wistar rats	half male and half female	NA	200-250	12/12	2VO	24	MA	GV24,GV20,GV21,EX-HN1,BL4,GB13,BL7,BL6,GB17,GB16	6h, once daily for 4 weeks	The needles were stimulated by twirling at 200 rpm for 3 minutes, once hourly.	/	Hippocampus	①③④	a
Ye et al. (2017) (Ye et al., 2017b)	Wistar rats	Male	NA	270-300	34/34	2VO	68	MA	GV20, ST36	Once daily for 2 weeks, 1 day of rest after six treatments	The needles were stimulated by twirling at 120 rpm for 30 seconds	Sham acupuncture	Hippocampus	③⑤	a
Zhang (2014) (Zhang et al., 2014)	Kunming mice	Male	NA	28-35	20/20	2VO	40	EA	GV14,GV20, BL17, ST36	10 min, once daily for 15 days	Dense-Sparse Wave, 2–80 Hz	/	Hippocampus	①②③	c, d

2VO, Two-Vessel Occlusion; SNP, Sodium nitroprusside; BCAS, Bilateral common carotid artery stenosis; EA, Electroacupuncture; MA, Manual Acupuncture. ①, 5-HT; ②, NE; ③, DA; ④, ACh; ⑤, LTP; a, MWM-Escape latency; b, MWM-Number of platform crossings; c, Step-Down Latency; d, Number of Step-Down Errors.

### 3.3 Risk of bias

**Figure 2:**
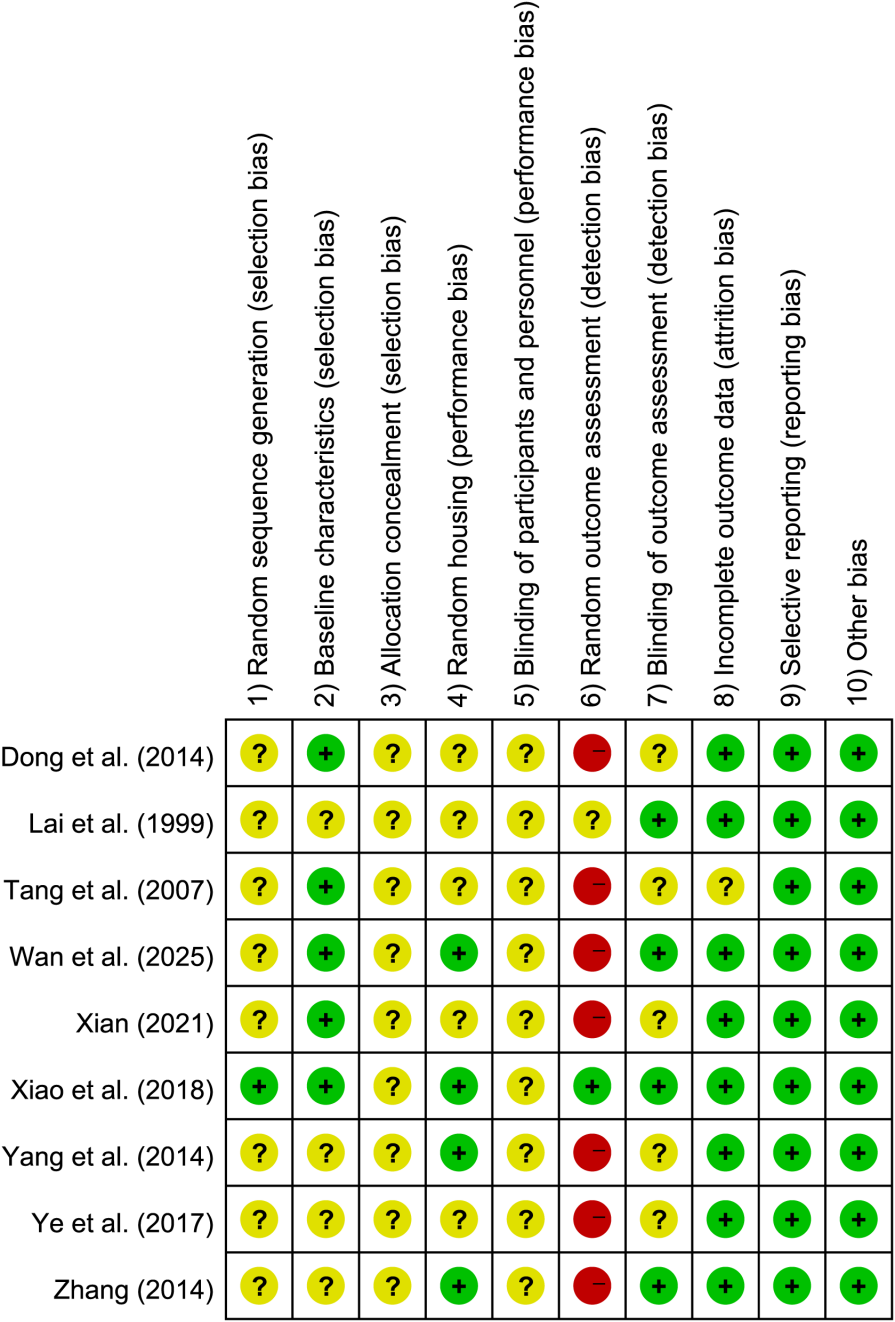
Risk of bias summary.

**Figure 3:**
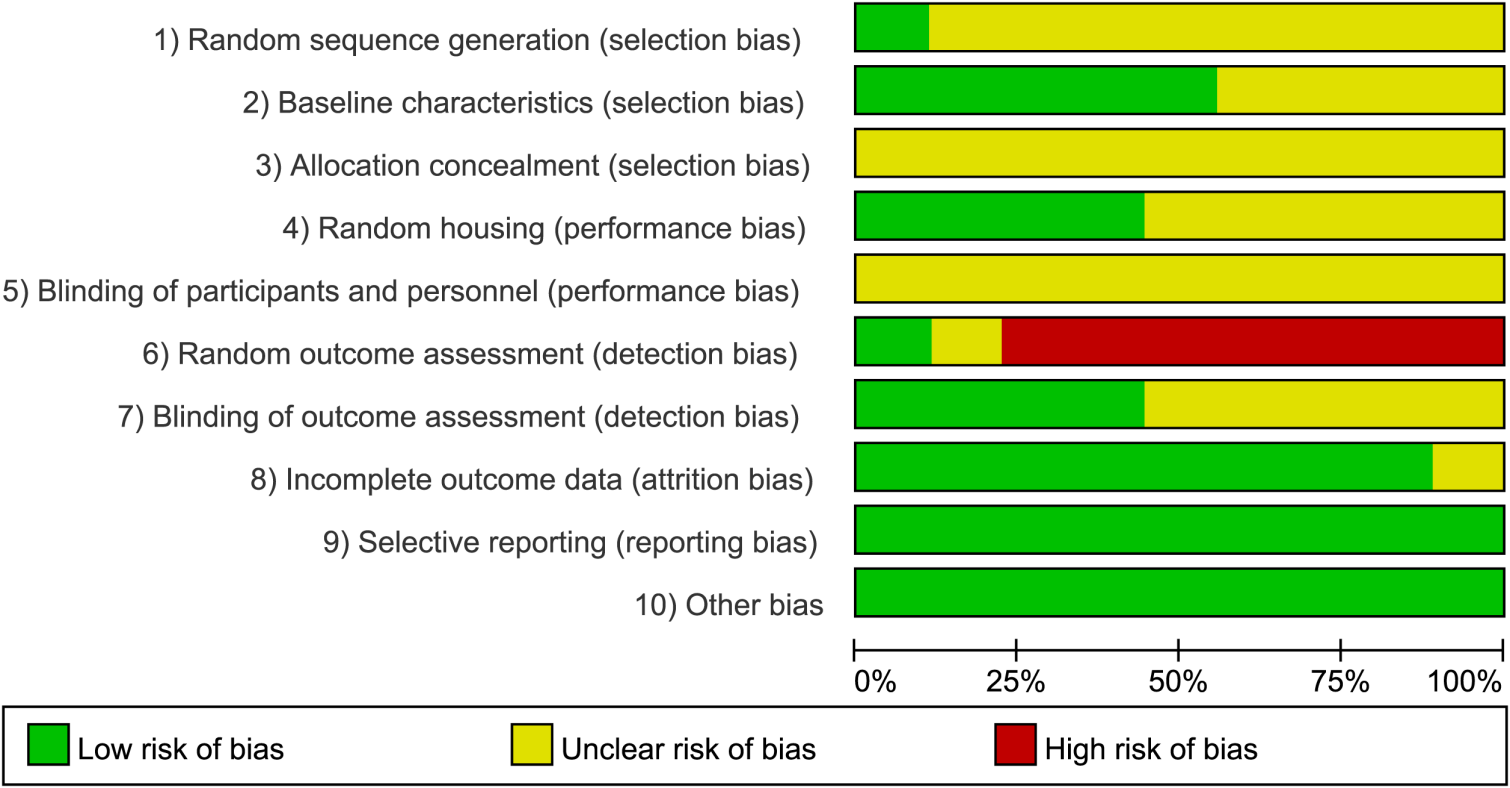
Risk of bias graph.

The potential risk of bias for the included animal studies was evaluated utilizing the SYRCLE tool, with the detailed results presented in **Figures 2**, **3**. Regarding random sequence generation (item 1), one study (Xiao et al., 2018) explicitly reported utilizing SPSS 20.0 software to generate random numbers for allocation and was consequently assigned a “low risk” rating. The remaining eight studies (Lai et al., 1999; Xian, 2004; Ming et al., 2007; Yang et al., 2014; Zhang, 2014; Juan et al., 2017; Ye et al., 2017a; Wan et al., 2025) failed to specify their randomization methods and were thus categorized as “unclear risk.” For baseline characteristics (item 2), five studies (Xian, 2004; Ming et al., 2007; Juan et al., 2017; Xiao et al., 2018; Wan et al., 2025) confirmed baseline balance and were evaluated as “low risk,” whereas the other four (Lai et al., 1999; Yang et al., 2014; Zhang, 2014; Ye et al., 2017a) lacked clear reporting on baseline equivalence, resulting in an “unclear risk” designation. Allocation concealment (item 3) was not described in any of the nine included studies, prompting an “unclear risk” rating across the board. Concerning random housing (item 4), four studies (Yang et al., 2014; Zhang, 2014; Xiao et al., 2018; Wan et al., 2025) documented randomized housing arrangements (“low risk”), while the remaining five omitted this methodological detail (“unclear risk”). For the blinding of caregivers and investigators (item 5), implementing adequate blinding was deemed methodologically unfeasible for MA and EA interventions; consequently, all studies were conservatively rated as “unclear risk.” In terms of random outcome assessment (item 6), one study (Xiao et al., 2018) explicitly employed a random number table for animal selection (“low risk”). Another study (Lai et al., 1999) was assigned an “unclear risk” due to the lack of specific randomization details, while the remaining seven studies lacked appropriate randomization in this aspect and were subsequently rated as “high risk.” Regarding the blinding of outcome assessors (item 7), four studies (Lai et al., 1999; Zhang, 2014; Xiao et al., 2018; Wan et al., 2025) were deemed “low risk” as their outcome measures were objective and ostensibly unaffected by the assessors. The other five studies (Xian, 2004; Ming et al., 2007; Yang et al., 2014; Juan et al., 2017; Ye et al., 2017a) provided insufficient information and were classified as “unclear risk.” For incomplete outcome data (item 8), one study (Ming et al., 2007) failed to adequately report the disposition of certain mice, yielding an “unclear risk.” The remaining eight studies presented complete datasets and were classified as “low risk.” Neither selective reporting (item 9) nor other sources of bias (item 10) were detected in any of the literature, meriting a “low risk” rating for all nine inclusions. Overall, the assessment revealed a generally low risk of bias concerning data completeness, baseline balance, and selective outcome reporting. Conversely, methodological deficiencies resulting in higher risks of bias were predominantly observed in random sequence generation, allocation concealment, random outcome assessment, and blinding procedures.

### 3.4 Primary outcomes

We selected monoaminergic neurotransmitters including 5-HT, NE, and DA as outcome measures for meta-analysis to evaluate the effects of acupuncture treatment on monoaminergic neurotransmitters in VaD models. The results are presented below:

#### 3.4.1 5-HT

**Figure 4:**
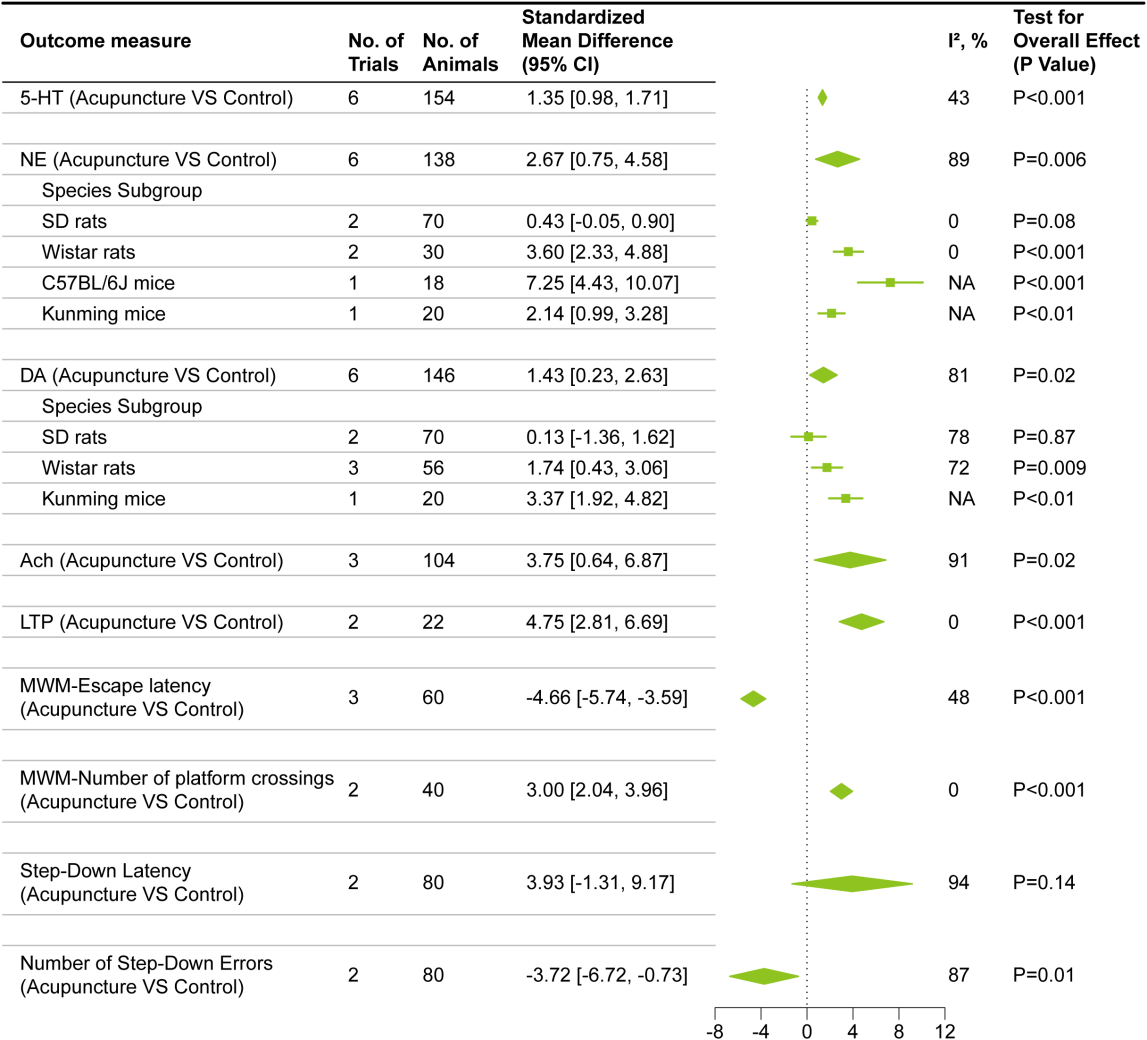
Forest plots of result of meta-analysis.

As illustrated in **Figure 4**, the pooled effect size for the neurotransmitter 5-HT encompassed six studies involving 154 rodents. Given the low heterogeneity observed across these studies (I²= 43%), a fixed-effects model was applied. The synthesized results revealed a statistically significant difference between the acupuncture and control groups (SMD = 1.35, 95% CI [0.98, 1.71]).

#### 3.4.2 NE

For the meta-analysis of NE, six studies comprising 138 rodents were evaluated. A high degree of heterogeneity was detected in the pooled effect size (I² = 89%). Subsequent sensitivity analysis (**Supplementary Material 2**) failed to resolve this substantial variance. Consequently, subgroup analyses were conducted utilizing animal species as the potential source of heterogeneity. Within the SD rat subgroup (two studies), heterogeneity was not significant; however, the random-effects model indicated no statistically significant difference between the acupuncture and control groups (SMD = 0.43, 95% CI [-0.05, 0.90]). Conversely, the Wistar rat subgroup (two studies) exhibited low heterogeneity (I²= 0%), and the random-effects model demonstrated a significant difference between the two groups (SMD = 3.60, 95% CI [2.33, 4.88]). In the subgroup using C57BL/6J mice, only one study was included, demonstrating a significant difference between the acupuncture group and the control group (SMD = 7.25, 95% CI [4.43, 10.07]). Similarly, in the subgroup using Kunming mice, only one study was included, which also showed a significant difference between the acupuncture group and the control group (SMD = 2.14, 95% CI [0.99, 3.28]) (**Figure 4**).

#### 3.4.3 DA

The meta-analysis for DA encompassed six studies involving 146 rodents. Similar to the NE findings, high heterogeneity was detected (I² = 81%), which persisted despite sensitivity analysis (**Supplementary Material 2**). Subgroup analysis based on animal species was therefore executed. In the SD rat subgroup (two studies), high heterogeneity remained (I²=78%), and a random-effects model revealed no significant difference between the treatment and control groups (SMD = 0.13, 95% CI [-1.36, 1.62]). In the Wistar rat subgroup (three studies), despite the persistence of high heterogeneity (I²=72%), the random-effects model indicated a statistically significant difference between the acupuncture and control groups (SMD = 1.74, 95% CI [0.43, 3.06]). The Kunming mice subgroup, consisting of a single study, also demonstrated a significant difference between the groups (SMD = 3.37, 95% CI [1.92, 4.82]) (**Figure 4**).

Collectively, these findings suggest that acupuncture interventions can effectively modulate the levels of monoaminergic neurotransmitters (5-HT, NE, and DA) in animal models of VaD.

### 3.5 Secondary outcomes

Secondary outcome measures included ACh, LTP, and behavioral assessments (Number of platform crossings and Escape latency in MWM, latency period and error frequency in Step-Down test), with specific results as follows:

#### 3.5.1 ACh

For the meta-analysis of ACh, three studies comprising 104 rodents were included. A high degree of heterogeneity was observed in the pooled analysis (I²=91%). As sensitivity analysis (**Supplementary Material 2**) failed to resolve this substantial variance, a random-effects model was applied. The synthesized results demonstrated a statistically significant difference between the acupuncture and control groups (SMD = 3.75, 95% CI [0.64, 6.87]) (**Figure 4**).

#### 3.5.2 LTP

The pooled effect size for LTP (**Figure 4**) encompassed two studies involving 22 rodents. Given that no statistical heterogeneity was observed (I²=0%), a fixed-effects model was employed. The analysis revealed a statistically significant difference between the acupuncture and control groups (SMD = 4.75, 95% CI [2.81, 6.69]).

#### 3.5.3 MWM-escape latency

For MWM-escape latency, four studies were initially evaluated, but substantial heterogeneity was detected (I²=87%). Subsequent sensitivity analysis (**Supplementary Material 2**) revealed a reduction in heterogeneity to I² = 48% following the exclusion of the study by Xian et al (Xian, 2004). Consequently, three studies comprising 60 rodents were ultimately retained. Utilizing a fixed-effects model, the pooled results demonstrated a significant reduction in escape latency in the acupuncture group compared to the control group (SMD = -4.66, 95% CI [-5.74, -3.59]) (**Figure 4**).

#### 3.5.4 MWM-number of platform crossings

Regarding the number of MWM platform crossings, two studies involving 40 rodents were analyzed. No heterogeneity was observed (I²=0%), and the application of a fixed-effects model indicated a statistically significant increase in crossings for the acupuncture group relative to the control group (SMD = 3.00, 95% CI [2.04, 3.96]).

#### 3.5.5 Step-down latency

The meta-analysis for Step-Down Latency included two studies involving 80 rodents. Due to the presence of extremely high heterogeneity (I²=94%), a random-effects model was utilized. Notably, the pooled results revealed no statistically significant difference between the acupuncture and control groups for this specific behavioral parameter (SMD = 3.93, 95% CI [-1.31, 9.17]).

#### 3.5.6 Number of step-down errors

For the Number of Step-Down Errors, two studies encompassing 80 rodents were evaluated. Significant heterogeneity was detected (I²=87%), necessitating the use of a random-effects model. The analysis identified a statistically significant reduction in error frequency within the acupuncture group compared to the control group (SMD = -3.72, 95% CI [-6.72, -0.73]) (**Figure 4**).

Collectively, the secondary outcome measures indicate that acupuncture can effectively modulate the neurotransmitter ACh, enhance synaptic plasticity (as evidenced by improved LTP), and behaviorally ameliorate cognitive impairment in VaD models. These behavioral improvements are robustly manifested through reduced escape latency and increased platform crossings in the MWM, alongside decreased error frequencies in the Step-Down test.

## 4 Discussion

### 4.1 Sources of heterogeneity

Before analyzing specific outcome measures, it is essential to clearly distinguish between the statistical heterogeneity observed in our analyses (reflected by high I^2^ values) and the underlying biological and methodological heterogeneity driving it. In this preclinical review, biological heterogeneity primarily stems from inherent variations in animal species (e.g., SD rats vs. Wistar rats) and the distinct pathophysiological mechanisms of different VaD modeling methods (e.g., 2VO vs. thromboembolic models). Concurrently, methodological heterogeneity arises from the diverse acupuncture intervention protocols employed across studies, including differences in modality (MA vs. EA), acupoint selection, and stimulation parameters (frequency, intensity, and duration). To provide a more structured analysis, we have systematically summarized these potential biological and methodological sources of heterogeneity in **Supplementary Material 4**. Given the limited number of available studies, these foundational variations inevitably manifest as substantial statistical heterogeneity in certain pooled outcomes.

For the primary outcome measure, 5-HT, the pooled analysis demonstrated low heterogeneity (I² = 43%) and a statistically significant effect size, suggesting that acupuncture can effectively regulate 5-HT levels in VaD animal models. However, given the methodological limitations of the included studies, these findings warrant cautious interpretation. We anticipate that future rigorously designed and high-quality animal studies will yield more robust evidence to confirm these therapeutic effects.

Conversely, the meta-analysis for NE exhibited substantial heterogeneity (I² = 89%). Initial sensitivity analysis failed to meaningfully reduce this variance (I² ≥ 84%), necessitating a more in-depth exploration of its sources. We sequentially hypothesized that the heterogeneity might stem from the specific acupuncture modality or the VaD modeling method. Subgroup analyses stratified by intervention type MA versus EA and modeling technique (e.g., 2VO, modified 2VO with SNP, BCAS, and cerebral embolism models) were conducted. However, substantial heterogeneity persisted within these subgroups despite sustained significant differences between the treatment and control groups, precluding definitive conclusions from these stratifications. Subsequent scrutiny of the baseline characteristics revealed considerable variation in the animal species utilized across the studies, encompassing SD rats, Wistar rats, C57BL/6J mice, and Kunming mice. Notably, subgroup analysis based on animal species successfully resolved the heterogeneity. Within both the SD rat and Wistar rat subgroups, heterogeneity was completely eliminated (I² = 0%). Although the pooled effect in the SD rat subgroup did not reach statistical significance, its trend aligned consistently with the other subgroups. Concurrently, statistically significant differences between the acupuncture and control groups were observed in the Wistar rat subgroup, as well as in the C57BL/6J and Kunming mice subgroups (which comprised only one study each). Consequently, we infer that the high heterogeneity observed in the NE effect sizes is primarily attributable to inter-species variations. Nevertheless, due to the limited number of available studies within each species-specific subgroup, this interpretation must be approached with caution and requires corroboration by future research.

Similarly, substantial overall heterogeneity (I² = 81%) was detected in the meta-analysis of the pooled effect sizes for DA. Sensitivity analysis revealed the persistence of this high heterogeneity (I² ≥ 76%), indicating significant outcome inconsistencies across the included studies. Unlike the findings for NE, subgroup analysis stratified by animal species did not yield a marked reduction in heterogeneity. Within the SD rat subgroup, the pooled effect size for DA failed to achieve statistical significance and was accompanied by high intra-group heterogeneity (I² = 78%), highlighting substantial variability among the individual studies. The Wistar rat subgroup similarly exhibited high heterogeneity (I² = 72%); however, a statistically significant difference was observed between the acupuncture and control groups, suggesting that acupuncture exerts potential regulatory effects on DA within this specific model. The Kunming mouse subgroup comprised only a single study, which, despite demonstrating a significant difference, provides limited evidentiary weight. Based on these findings, we postulate that the sources of heterogeneity for the DA indicator are multifactorial, potentially intertwining with modeling methodologies and specific acupuncture intervention modalities, rather than being solely attributable to animal species. Specifically, the SD rat subgroup encompassed only two studies with divergent intervention modalities: the study by Lai et al. utilized EA at a specific frequency of 10–12 Hz, whereas the study by Tang et al. employed MA, which intrinsically lacks an equivalent, quantifiable high-frequency stimulation pattern. Extant research indicates that the rapid release of DA relies heavily on presynaptic Ca²^+^ sensors (e.g., the synaptotagmin family) to fine-tune the spatiotemporal dynamics of Ca²^+^ signaling in response to specific stimulation frequencies. Concurrently, DA-related synaptic plasticity exhibits differential responses to varying stimulation frequencies, strongly implying a frequency-dependent regulatory mechanism (Nakano et al., 2010; Banerjee et al., 2020). EA utilizes controllable and quantifiable frequency parameters, whereas MA is characterized by variable and unstandardized stimulation patterns. Consequently, this inherent methodological divergence may largely account for the substantial inter-study variability observed in DA modulatory effects. Furthermore, within the Wistar rat subgroup, the study by Xian et al. employed a cerebral embolization model, whereas the studies by Yang et al. and Ye et al. utilized the 2VO model, introducing significant pathological variance between the two approaches. Subsequent subgroup analysis stratified by these modeling methods revealed an absence of heterogeneity between the studies by Yang et al. and Ye et al., indicating that the intra-group variance primarily originated from Xian et al.’s embolization model. This finding substantiates the inference that methodological differences in VaD modeling constitute another critical source of the observed heterogeneity.

Similarly, a high degree of overall heterogeneity was observed in the meta-analysis for the outcome measure of ACh (I² = 91%), which persisted despite rigorous sensitivity analyses (I² ≥ 84%). Further scrutiny of the study characteristics suggests that this substantial variance may be primarily attributable to the diversity of VaD model types and their distinct patterns of cholinergic system impairment. Specifically, the meta-analysis for ACh encompassed three studies utilizing three disparate modeling methodologies: the 2VO model, the modified 2VO combined with SNP model, and the cerebral embolization model. These diverse models exhibit varying degrees of structural damage to the basal forebrain-cortical cholinergic pathway, which serves as the critical anatomical foundation for ACh synthesis, release, and functional activity (Nishio et al., 2010; Xu et al., 2020; Mirza Agha et al., 2021). Consequently, the magnitude and trajectory of acupuncture-induced ACh modulation may fluctuate significantly across these different pathological contexts, thereby amplifying inter-study inconsistencies. Furthermore, the regulation of ACh is highly contingent upon the structural integrity of cholinergic neurons, alongside the activity of specific enzymes governing its synthesis and degradation. It is postulated that acupuncture exerts an indirect, multi-level modulatory influence on ACh levels (Richter et al., 2018; Bekdash, 2021). This mechanistic complexity renders ACh indicators exceptionally susceptible to variations in animal species, modeling severity, and broader experimental conditions. Therefore, the observed high heterogeneity more likely reflects inherent pathological and methodological divergences rather than true inconsistencies in the therapeutic efficacy of the acupuncture intervention itself.

Regarding the outcome measure of LTP, the pooled analysis incorporated two studies and revealed a complete absence of heterogeneity (I² = 0%). Moreover, a statistically significant difference was identified between the acupuncture and control groups. This outcome measure provides robust electrophysiological evidence substantiating acupuncture’s potential to ameliorate cognitive impairment and restore synaptic plasticity in VaD models.

Regarding the behavioral assessments, four specific metrics were evaluated, encompassing two parameters from the MWM test: escape latency and the number of platform crossings. For MWM-Escape latency, the initial inclusion of four studies yielded extremely high heterogeneity (I² = 87%). However, sensitivity analysis demonstrated that excluding the study by Xian et al. substantially reduced this variance (I² = 48%) and revealed a statistically significant difference between the acupuncture and control groups. We attribute this heterogeneity to the inherent pathological discrepancies between the cerebral embolization model utilized by Xian et al. and the 2VO-based models employed in the remaining three studies. Extant research indicates that in cerebral embolization models, unless the confounding influence of motor deficits is strictly disentangled from behavioral parameters, prolonged escape latencies in the MWM cannot be reliably interpreted as a pure reflection of cognitive impairment (Bingham et al., 2012). Conversely, the meta-analysis for the number of platform crossings incorporated only two studies and detected zero heterogeneity (I² = 0%), demonstrating a statistically significant improvement in the acupuncture group. In addition to spatial memory evaluation via the MWM, this review incorporated two metrics from the Step-Down test to assess passive avoidance learning and memory capabilities. For Step-Down Latency, the pooled analysis of two studies exhibited extremely high heterogeneity (I² = 94%) and failed to show a statistically significant difference between the acupuncture and control groups.

It is crucial to recognize that, unlike the MWM, behavioral performance in the Step-Down test is modulated not solely by learning and memory capacity, but also by confounding physiological factors such as the animal’s emotional state, pain sensitivity, and stress responses. Furthermore, discrepancies in modeling methodologies and specific experimental parameters (e.g., electric shock intensity and training protocols) can substantially alter avoidance behavior strategies (Kameyama et al., 1986; López-Moraga et al., 2022). Consequently, the profound heterogeneity observed for this indicator is more likely a reflection of divergent behavioral paradigms and varying assessment sensitivities, rather than a true inconsistency in the cognitive modulatory efficacy of the acupuncture intervention. Similarly, the meta-analysis for the Number of Step-Down Errors (two studies) revealed extremely high heterogeneity (I² = 87%); nevertheless, a statistically significant difference was still detected between the treatment and control groups. We postulate that the primary sources of heterogeneity for this metric align directly with those identified for Step-Down Latency. Ultimately, given the limited number of studies currently available for these behavioral subgroup analyses, interpretation of these functional outcomes mandates caution, and the conclusions drawn herein await robust validation by future comprehensive investigations.

### 4.2 Multi-pathway regulation of monoaminergic neurotransmitters by acupuncture

Drawing upon the meta-analysis results of the present study, it can be inferred that acupuncture interventions effectively modulate the levels of monoaminergic neurotransmitters in VaD animal models. However, the current body of evidence is insufficient to delineate whether this modulatory effect primarily manifests as transient fluctuations in neurotransmitter release or represents a fundamental recalibration of the neurochemical milieu following repeated interventions. Given that monoaminergic systems predominantly exert systemic neuromodulatory functions, their alterations are more likely to reflect global shifts in neural network states rather than the direct encoding of specific cognitive information (Azizi, 2022; Özçete et al., 2024). In VaD models, chronic cerebral hypoperfusion precipitates the functional dysregulation of the monoaminergic system, thereby significantly impairing the adaptive capacity of neural circuits in response to external stimuli and synaptic activity (Tao et al., 2014; Xiao et al., 2017; Zhang, 2022). Consequently, acupuncture may facilitate the restoration of systemic monoaminergic homeostasis, thereby cultivating a more permissible neurochemical environment for subsequent synaptic remodeling and functional recovery. It is noteworthy that neurotransmitter aberrations in VaD are not confined to the monoaminergic system; cholinergic dysfunction is concurrently recognized as a hallmark pathological feature (Noufi et al., 2019). Previous literature delineates a profound functional coupling between these systems. Specifically, DA, NE, and 5-HT can indirectly modulate neural network dynamics associated with attention, learning, and synaptic plasticity by regulating the excitability and neurotransmitter release profiles of cholinergic neurons in the basal forebrain and cortex (Švob Štrac et al., 2016; Hyun Lee et al., 2025; Liu et al., 2025). Thus, in the context of chronic cerebral hypoperfusion, monoaminergic dysregulation may synergistically amplify the deleterious impacts of cholinergic deficits on neural network adaptability. Acupuncture’s regulation of the monoaminergic system may, therefore, orchestrate cognitive restoration through the enhancement of monoaminergic-cholinergic co-modulation. This mechanistic inference is consistent with our meta-analysis findings, which indicate a statistically significant elevation in ACh levels following acupuncture treatment relative to the control group.

Mechanistically, acupuncture regulates the monoaminergic system not through a singular target, but via a highly integrated, multi-level, and multi-pathway neuromodulatory network (Xu et al., 2013). On one hand, mechanical stimulation at key acupoints (e.g., GV20, ST36, and GV24) activates densely distributed sensory receptors and peripheral nerve endings. This mechanical-to-neural signal transduction propagates via peripheral sensory afferents to spinal cord and brainstem nuclei, thereby initiating central regulatory cascades associated with the “skin-brain axis” (He et al., 2025). These ascending signals are hypothesized to activate critical monoaminergic nuclei, including the locus coeruleus (LC), ventral tegmental area (VTA), and dorsal raphe nucleus (DRN), thereby potentially augmenting the firing activity of NE, DA, and 5-HT neurons and facilitating the modulation of their corresponding neurotransmitter levels (Yu et al., 2024; Pan et al., 2025; Wan et al., 2025). Furthermore, extensive preclinical evidence indicates that acupuncture upregulates the expression and catalytic activity of rate-limiting synthetic enzymes, such as tyrosine hydroxylase (TH) and tryptophan hydroxylase (TPH) (Guo et al., 2024; Chen et al., 2025), thereby facilitating the synthesis and subsequent release of these monoamines. Concurrently, acupuncture has been reported to modulate the functional state of monoamine transporters, such as the norepinephrine transporter (NET), dopamine transporter (DAT), and serotonin transporter (SERT), rectifying imbalances in neurotransmitter reuptake. This regulatory action may curtail excessive synaptic clearance and degradation rates, thereby prolonging the effective temporal window of neurotransmitters within the synaptic cleft and sustaining their modulatory efficacy (Cao et al., 2011; Cai et al., 2023; Ma et al., 2023). Aligning with these proposed multi-target mechanisms, specific studies within our meta-analysis provide supportive *in vivo* evidence. The study by Wan et al. (2025) demonstrated that in VaD models, EA robustly activates the “LC-PFC circuit,” promoting NE release and providing direct empirical support for the “LC monoaminergic nucleus activation” hypothesis. Similarly, the study by Ye et al. (2017a) established that acupuncture enhances DA levels and exerts its effects via the DA-D1/D5 receptor pathway; given that DA is predominantly synthesized and released by VTA neurons, this finding indirectly but strongly corroborates the “activation of the VTA monoaminergic nucleus” mechanism.

On the other hand, acupuncture preserves the structural and functional integrity of monoaminergic neurons through multifaceted neuroprotective mechanisms. In the context of chronic cerebral hypoperfusion and vascular injury, the study by Lai et al. (1999), included in this meta-analysis, demonstrated that acupuncture enhances superoxide dismutase (SOD) activity in VaD animal models. Similarly, the study by Zhang et al (Zhang, 2014). revealed that acupuncture increases hippocampal SOD activity while reducing malondialdehyde (MDA) content in VaD mice. These findings indicate that EA bolsters the endogenous capacity to scavenge reactive oxygen species (ROS), attenuating the excessive ROS accumulation induced by chronic hypoperfusion and mitigating ROS-mediated neuronal damage (Iadecola, 2013). Concurrently, the study by Xian et al (Xian, 2004). indicates that acupuncture techniques can reduce the accumulation of excitatory amino acids, such as glutamate and aspartate, in the hippocampus, cortex, and striatum of VaD rats. By antagonizing excitotoxicity, this intervention decreases the apoptosis of monoaminergic neurons, protects nerve fiber density in the hippocampus and PFC, and helps prevent the decline in neurotransmitter synthesis capacity. Moreover, recent literature highlights neuroinflammation as a pathological factor contributing to the disruption of monoaminergic homeostasis (Saggu et al., 2025). Sustained inflammatory responses disrupt the synthesis, release, and receptor signaling of NE, 5-HT, and DA via the secretion of inflammatory cytokines and the aberrant activation of glial cells, thereby impairing monoaminergic neurotransmission (Saggu et al., 2025). In turn, the depletion of monoaminergic neurotransmitters may exacerbate neuroinflammation, creating a detrimental cycle. Consequently, mitigating neuroinflammation is generally regarded as an essential prerequisite for re-establishing monoaminergic homeostasis. Accumulating evidence indicates that acupuncture can modulate neuroinflammation in VaD animal models, thereby ameliorating corresponding behavioral deficits (Li et al., 2022; Wen et al., 2023). By suppressing neuroinflammation, acupuncture attenuates the pathological negative coupling between inflammatory cascades and the monoaminergic system, providing important support for the restoration of neurotransmitter equilibrium.

At the macroscopic level of the cerebral microenvironment, acupuncture mitigates chronic cerebral hypoperfusion by enhancing regional cerebral blood flow (CBF) and metabolic supply in cortical areas (Zhang et al., 2014). Among the studies included in our review, the research by Lai et al. (1999) demonstrated that EA improves CBF and elevates monoamine neurotransmitter levels in the cerebral cortex, phenomena that are accompanied by the recovery of learning and memory capabilities. This suggests that by optimizing cerebral perfusion and the metabolic microenvironment, acupuncture provides a physiological foundation for restoring monoaminergic system function. Furthermore, the study by Wan et al. (2025) elucidated the underlying mechanisms at the neural circuit level: EA activates the LC-PFC-NE pathway, augments CBF, and restores neuronal activity. This provides circuit-level validation of acupuncture’s synergistic regulation of both CBF and the monoaminergic system. Building upon this physiological foundation, acupuncture may further enhance the efficacy of monoamine neurotransmitters by modulating key downstream signaling cascades. Mechanisms identified within our included literature involve facilitating the modulatory release of NE within the LC-PFC pathway, or enhancing postsynaptic responses via signaling pathways such as DA-D1/D5 receptors and NE-β_1_-AR. These actions collectively improve the functional efficiency of monoaminergic neurotransmitters in facilitating synaptic plasticity, neural network integration, and behavioral regulation (Ye et al., 2017a; Xiao et al., 2018; Wan et al., 2025). Crucially, certain included studies reported no statistically significant differences in monoamine levels between the acupuncture group and healthy control counterparts, embodying the principle of “restoring equilibrium rather than inducing excessive elevation.” This finding indicates that the regulatory action of acupuncture is primarily geared toward re-establishing the dynamic homeostasis and modulatory capacity of the monoaminergic system, rather than inducing non-physiological increases in neurotransmitter concentrations.

### 4.3 Monoaminergic neurotransmitter-mediated modulation of synaptic plasticity

At the synaptic level, LTP is widely recognized as a fundamental cellular substrate for learning and memory formation. Its induction and maintenance processes are highly sensitive to the surrounding neurochemical milieu (Shors and Matzel, 1997). As demonstrated in previous studies, the expression of LTP relies not only on the synchronized activity of pre- and postsynaptic neurons but is also dynamically governed by the background regulation of multiple neuromodulatory systems (Jahan et al., 2026). An accumulating body of evidence indicates that monoaminergic neurotransmitters do not directly trigger rapid synaptic events; rather, they reshape neuronal responses to activity-dependent stimuli by regulating key molecular pathways, ultimately modulating overall synaptic plasticity and adjusting the threshold for LTP induction. These proposed regulatory mechanisms encompass the dynamic modulation of N-methyl-D-aspartate (NMDA) receptor function (e.g., enhancing its activity via potassium channel inhibition, direct phosphorylation, or astrocyte-mediated D-serine release), alongside the potential regulation of α-amino-3-hydroxy-5-methyl-4-isoxazolepropionic acid (AMPA) receptor trafficking and membrane insertion, cooperatively driving the critical Ca^2+^ and Na^+^ influxes required for synaptic remodeling. Furthermore, these processes engage intracellular signaling networks centered on the cyclic adenosine monophosphate (cAMP)/protein kinase A (PKA) cascade, cAMP response element-binding protein (CREB)-mediated gene transcription, and calcium signaling pathways. This multidimensional molecular framework elucidates how monoaminergic neurotransmitters facilitate the enhancement of synaptic plasticity (O’Dell et al., 2015; Palacios-Filardo and Mellor, 2019; Azizi, 2022).

It is crucial to note that while our meta-analysis provides direct evidence for the regulatory effects of acupuncture on monoamine levels and LTP in VaD models, the specific involvement of the aforementioned downstream molecular cascades is largely extrapolated from the broader neurobiological literature. Therefore, these proposed pathways represent highly plausible mechanistic hypotheses bridging neurotransmitter restoration to synaptic plasticity, rather than direct empirical conclusions derived from the current dataset.

NE primarily originates from the LC and bidirectionally modulates synaptic excitability and the cAMP/PKA signaling pathway through the activation of α and β adrenergic receptors. (predominantly the β_1_ and β_2_ subtypes). Simultaneously, NE modulates NMDA and AMPA receptor functions and inhibits K^+^ channels, thereby enhancing dendritic depolarization. Its release kinetics (e.g., transient burst activation versus sustained tonic release) and concentration fluctuations are thought to exert a bidirectional modulatory effect on LTP, capable of either facilitating or suppressing it to dynamically influence synaptic plasticity (O’Dell et al., 2015).

DA is predominantly synthesized in the VTA. Its receptors are broadly categorized into D1-like (D1 and D5) and D2-like (D2, D3, and D4) families, which respectively upregulate and downregulate intracellular cAMP levels to bidirectionally modulate synaptic plasticity (Palacios-Filardo and Mellor, 2019). Generally, D1 receptors appear to exert the most profound influence on plasticity processes. Upon activation, D1 receptors mediate cAMP/PKA signaling cascades via Gs proteins. This cascade enhance postsynaptic NMDA receptor function and calibrate the inhibitory-excitatory balance of local microcircuits, thereby lowering the threshold for LTP induction. Concurrently, these signaling events also phosphorylate and activate CREB. This activation subsequently initiates gene transcription programs essential for synaptic structural remodeling and the maintenance of long-term plasticity (Hammad and Wagner, 2006; Varela et al., 2009).

5-HT originates from the DRN and governs hippocampal LTP and synaptic plasticity through receptor subtype-specific pathways. Functionally, its diverse receptors can be classified into three core modulatory categories: 5-HT_1_ and 5-HT_5_ receptors couple to Gi/Go proteins, inhibiting neuronal activity by reducing intracellular cAMP levels and increasing K^+^ conductance, while simultaneously engaging the CREB-1 transcription factor for gene regulation; 5-HT_2_, 5-HT_4_, 5-HT_6_, and 5-HT_7_ receptors couple to Gq and Gs proteins, elevating cAMP and Ca²^+^ concentrations to exert excitatory effects; and the 5-HT_3_ receptor functions as an ionotropic ligand-gated ion channel, directly inducing depolarization to enhance neuronal excitability and promote neurotransmitter release (Azizi, 2022).

Consequently, in VaD models, the restoration of monoaminergic neurotransmitter levels and their corresponding modulatory capacities may cultivate a more permissible neurochemical milieu for learning-related synaptic remodeling by lowering the LTP induction threshold and reinforcing its stability. As demonstrated in our meta-analysis, acupuncture effectively modulates neurotransmitter release in VaD animal models while concurrently influencing LTP expression. Among the included literature, the study by Ye et al. (2017a) demonstrated that acupuncture upregulates DA release and modulates synaptic plasticity in the hippocampal dentate gyrus (DG) region via the DA-D1/D5 receptor pathway. Similarly, the study by Xiao et al. (2018) indicated that acupuncture increases NE expression and impacts synaptic plasticity in the hippocampal DG through β_1_-adrenergic receptors (β_1_-AR). Furthermore, a previous study on EA treatment for ischemic stroke (Wang et al., 2020) revealed that the intervention downregulates the aberrant expression of 5-HT_1_A receptors. This downregulation reduces the inhibitory binding of 5-HT to 5-HT_1_A receptors, thereby disinhibiting PKA activity, rescuing hippocampal synaptic plasticity, and ultimately ameliorating learning and memory deficits post-stroke.

Collectively, these findings suggest that acupuncture may exert its cognitive-enhancing effects in VaD and post-ischemic stroke models by systematically regulating the release of key monoamines (DA, NE, and 5-HT) and calibrating their receptor expression profiles, thereby activating downstream signaling cascades such as PKA and consolidating hippocampal synaptic plasticity.

### 4.4 Synaptic plasticity and behavioral performance

The enhancement of LTP signifies improved synaptic plasticity, which typically correlates with enhanced learning and memory functions. However, behavioral task performance is not a direct, linear reflection of these microscopic synaptic events (Martin et al., 2000; Mukhtar and Iftikhar, 2025). Commonly employed cognitive-behavioral paradigms, such as the MWM and Step-Down tests, depend not only on learning and memory pathways but are also heavily influenced by multiple non-cognitive physiological factors, including emotional state, motivational drive, arousal levels, and stress responses. This phenomenon is particularly pronounced in chronic brain injury models such as VaD (Kameyama et al., 1986; Jung et al., 2017; López-Moraga et al., 2022).

The monoaminergic neurotransmitter system occupies a pivotal translational position bridging these micro- and macroscopic levels. Specifically, DA, NE, and 5-HT not only participate in the modulation of synaptic plasticity but also extensively regulate broader behavioral states, such as emotion, motivation, and arousal, thereby profoundly influencing the animals’ active engagement and response strategies during behavioral tasks (Avery and Krichmar, 2017).

Consequently, acupuncture may exert a dual modulatory effect via the monoaminergic system: on one hand, by optimizing the neurochemical milieu to foster favorable conditions for synaptic plasticity, which indirectly benefits cognitive performance; on the other hand, by stabilizing emotional and arousal states, thereby mitigating the interference of non-cognitive confounders on functional outcomes. Thus, the behavioral improvements observed following acupuncture intervention likely reflect both the enhancement of synaptic plasticity potential and the systemic modulation of behavioral states, rather than a simple, direct transformation of synaptic changes into task performance.

In the present study, our synthesized behavioral data indicate that acupuncture effectively reduces escape latency and increases the number of platform crossings in the MWM test. Furthermore, data from the Step-Down test (specifically the decreased number of errors) corroborate that acupuncture ameliorates behavioral deficits in VaD models. These macroscopic functional improvements are highly consistent with the neurochemical and electrophysiological results observed in our neurotransmitter and LTP analyses, solidifying the multi-target therapeutic profile of acupuncture.

### 4.5 Potential Mechanisms

**Figure 5:**
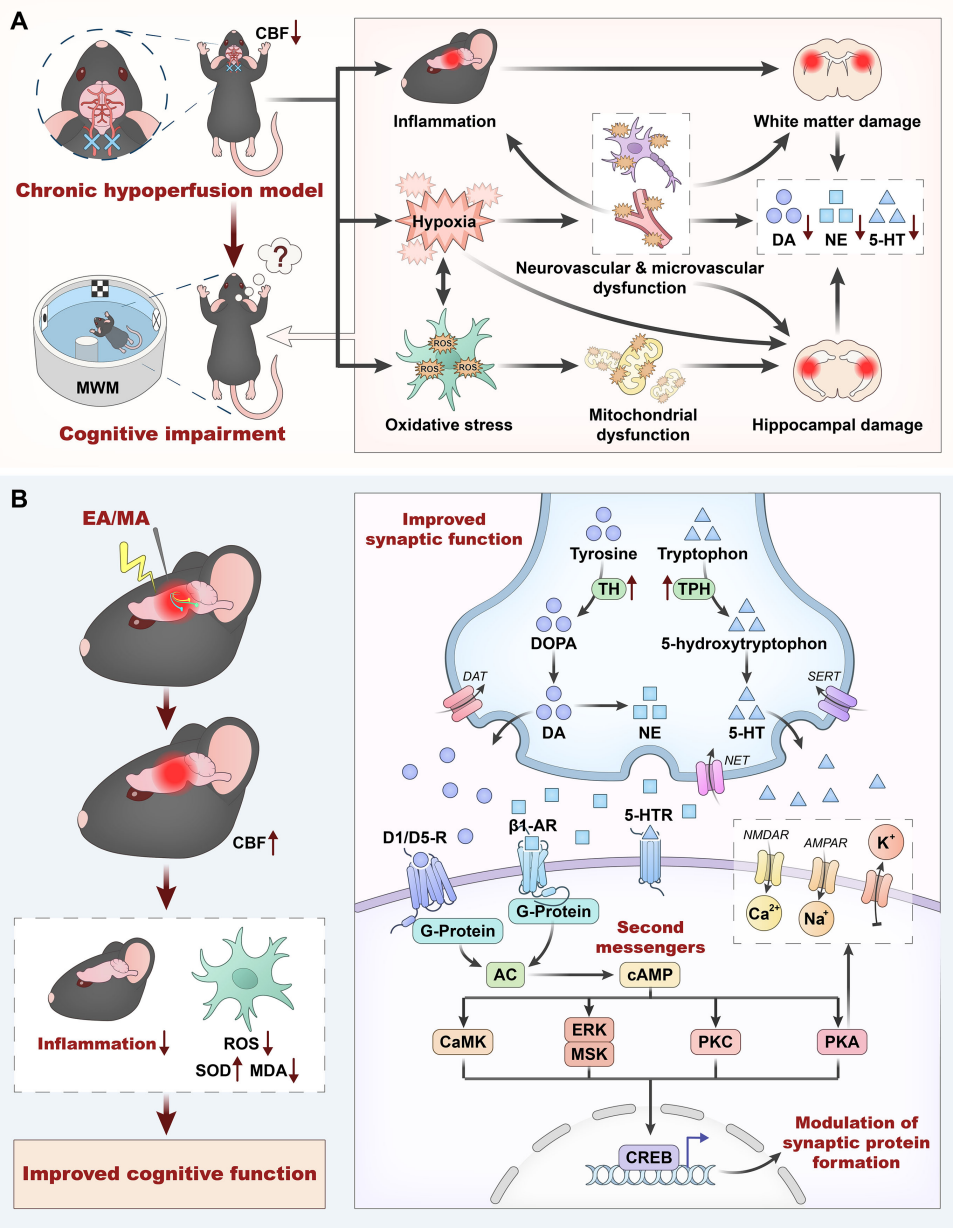
Potential mechanisms of acupuncture regulating monoaminergic neurotransmitters to improve cognitive function in VaD.

Based on the meta-analysis results of this study and existing literature, we have outlined and proposed a potential pathophysiological mechanism model of acupuncture’s multi-target modulation of monoamine neurotransmitters, improvement of synaptic plasticity, and ultimate restoration of cognitive function in VaD (**Figure 5**). As shown in **Figure 5A**, the pathological evolution of VaD is a cascade process initiated by abnormal CBF (Venkat et al., 2015; Durgapal et al., 2025). Cerebral vascular occlusion or dysfunction leads to sustained hypoperfusion in key brain regions, which not only deprives neurons of nutritional supply but also triggers the activation of oxidative stress and inflammatory pathways (Venkat et al., 2015). The disruption of the neurovascular microenvironment exacerbates local hypoxia and microcirculatory disturbances, amplifying the inflammatory response and leading to structural damage in the hippocampus and white matter (Chen et al., 2011; Johnson, 2023). Hypoxia-induced accumulation of ROS causes mitochondrial dysfunction, resulting in hippocampal neuronal apoptosis and impaired synaptic plasticity (Luca et al., 2015); concurrently, elevated inflammatory factors exacerbate white matter demyelination and axonal damage (Tian et al., 2022; Qneibi et al., 2024). These pathological factors intertwine, impeding neural signal transduction pathways via white matter damage (Bells et al., 2017) while directly affecting learning and memory-related neural circuits through hippocampal damage (Fortin et al., 2002), ultimately culminating in typical VaD symptoms such as cognitive decline and mood abnormalities. Furthermore, this cascade process leads to the dysregulation of monoaminergic neurotransmitter systems, including NE, DA, and 5-HT. The decline in neurotransmitter levels is not merely a consequence of tissue damage but constitutes a crucial component of the pathological vicious cycle in VaD (Court and Perry, 2003; Durgapal et al., 2025).

Targeting the aforementioned pathological cascades, existing evidence suggests that acupuncture possesses a systemic, multi-target interventional potential (as shown in **Figure 5B**). At the macroscopic and overall microenvironmental levels, acupuncture (EA/MA) may not only help reverse the chronic hypoperfusion state and increase CBF but also exhibit potential effects in mitigating neuroinflammation. Simultaneously, acupuncture may effectively scavenge ROS by enhancing SOD activity and reducing MDA levels, thereby blocking continuous oxidative stress-induced neuronal damage and fundamentally assisting in the restoration of the VaD microenvironment.

The amelioration of the microenvironment provides a physiological basis for the restoration of the neurotransmitter system and synaptic function. Regarding the specific pathways by which acupuncture modulates monoaminergic nuclei, the study by Wan et al. provides direct experimental evidence demonstrating that EA can effectively activate the LC-PFC neural circuit and promote NE release from the LC, with the activation of this pathway synchronously enhancing CBF. Based on this direct evidence, the meta-analysis results of the present study, and external supporting literature, we hypothesize that the restorative effect of acupuncture on monoaminergic nuclei might be systemic: in addition to the LC, it may also potentially enhance the functional status of the VTA and DRN. It is speculated that enhanced VTA activity could be a potential driver for the upregulation of DA levels, while improved DRN function might be accompanied by an increase in 5-HT content. Furthermore, drawing upon broader preclinical evidence, it is inferred that acupuncture may upregulate the levels of TH and TPH to promote neurotransmitter synthesis and release. Concurrently, it may inhibit excessive reuptake by modulating the functional status of transporters (NET, DAT, SERT), thereby potentially prolonging the effective duration of corresponding monoaminergic neurotransmitters in the synaptic cleft. Ultimately, these neurotransmitters are projected to reach the hippocampus via neural fiber circuits to exert their targeted modulatory effects.

Within the hippocampal synaptic regulatory network, upon the binding of NE to β_1_-ARs, G proteins transition from an inactive to an active state, activating adenylate cyclase (AC) (O’Dell et al., 2015). This facilitates the cAMP/PKA pathway to modulate the functions of NMDA and AMPA receptors, thereby influencing Ca²^+^ and Na^+^ influx. Simultaneously, by modulating the activity of specific types of K^+^ channels, it reduces the propensity for K^+^ efflux, enhances postsynaptic membrane depolarization, synergistically lowers the threshold for LTP, and regulates LTP expression in regions such as the hippocampal DG and CA1. Following its binding to D1/D5 receptors, DA similarly activates primarily the cAMP/PKA pathway to modulate NMDA and AMPA receptors, affecting Ca²^+^ and Na^+^ influx, and maintains the postsynaptic depolarized state by regulating K^+^ channel activity, thereby reducing the difficulty of LTP induction. Regarding 5-HT, although its receptor binding profile in VaD models remains incompletely elucidated, relevant evidence suggests that acupuncture may downregulate the aberrant expression of 5-HT_1_A receptors and elevate PKA signaling, influencing the postsynaptic membrane threshold to improve synaptic plasticity (given the multiplicity of 5-HT receptor subtypes, the specific mechanisms by which acupuncture modulates 5-HT binding to corresponding receptors to enhance synaptic plasticity in VaD models warrant further investigation).

It should be noted that the mechanisms by which monoamine neurotransmitters improve synaptic plasticity do not solely rely on the cAMP/PKA pathway. As depicted at the bottom of **Figure 5B**, CREB acts as a crucial transcriptional integration node that can be phosphorylated and activated by various upstream kinases. In addition to the cAMP-mediated PKA pathway, these include signaling pathways such as calcium/calmodulin-dependent protein kinase (CaMK), extracellular signal-regulated kinase/mitogen- and stress-activated protein kinase (ERK/MSK), and protein kinase C (PKC), which are mediated by Ca²^+^ or mitogen-activated protein kinase (MAPK) cascades, thereby initiating gene transcription associated with synaptic structural remodeling and the maintenance of long-term plasticity. These kinases can also collaboratively participate in the formation and maintenance of synaptic plasticity through post-translational modifications of synaptic proteins and the regulation of local protein synthesis processes (Mayr and Montminy, 2001; Azizi, 2022). The potential improvement in synaptic plasticity is ultimately reflected as enhanced cognitive function in behavioral outcomes.

However, despite this multidimensional modulatory mechanism providing a highly compelling theoretical framework, it must be acknowledged that current preclinical evidence is constrained by the methodological limitations of the included studies. In particular, the included studies generally exhibit a high risk of bias regarding allocation concealment and the implementation of blinding, which may exert a certain impact on the estimation of effect sizes. Therefore, interpretations of these mechanistic pathways should be approached with caution, and their exact causal relationships and potential translational clinical value remain to be further verified by more rigorously designed experimental studies in the future.

## 5 Strength

Although a previous systematic review and meta-analysis published in Chinese reported the regulatory effects of acupuncture on monoaminergic neurotransmitters in VaD animal models (Hou et al., 2022), that study primarily focused on pooling effect sizes without systematically elucidating the underlying neurobiological pathways, and its sample of included literature was relatively limited. Concurrently, while other meta-analyses have investigated the regulation of oxidative stress and inflammatory factors by acupuncture in VaD models (Wen et al., 2023; Bao et al., 2024), the specific role of the monoaminergic system warrants distinct attention. Monoaminergic neurotransmitters are widely recognized as a crucial neuromodulatory system integral to regulating synaptic plasticity and facilitating cognitive recovery, a premise supported by an accumulating body of preclinical evidence.

Building upon this foundation, the present study conducted a systematic retrieval and rigorous screening of the literature, ultimately incorporating nine animal experiments into the meta-analysis. By synthesizing evidence regarding acupuncture’s modulation of monoaminergic neurotransmitters, this study further explored the potential associations between these neurochemical alterations, enhancements in LTP, and behavioral improvements. However, given the substantial methodological and statistical heterogeneity observed among the included studies, these conclusions must be interpreted with caution. Nonetheless, this research provides a valuable theoretical framework and reference point for designing future rigorous animal experiments and advancing the mechanistic exploration of acupuncture in VaD.

## 6 Limitation

Through systematic review and meta-analysis, this study investigated the effects of acupuncture on monoaminergic neurotransmitters and related indicators in VaD animal models. While providing consolidated evidence for this field, several limitations may affect the reliability and generalizability of the results, as follows (1): Literature retrieval and publication-related biases. Although this study systematically searched eight major databases in both Chinese and English, the language restriction (only included studies in Chinese or English) inevitably excluded relevant studies published in other languages (such as Japanese, Korean, and German), which may have been indexed in regional databases (e.g., J-STAGE, KCI, LILACS). Furthermore, our literature search was restricted to officially published studies and did not encompass grey literature (such as unpublished dissertations, conference abstracts, and scientific reports). Given that studies with negative findings are less likely to be published, this omission inherently increases the risk of publication bias, potentially affecting the completeness of the included evidence. Notably, we were unable to quantitatively verify this bias; according to the Cochrane Handbook for Systematic Reviews of Interventions (Higgins et al., 2024), tests for funnel plot asymmetry (such as Egger’s or Begg’s tests) and the generation of funnel plots are not recommended when fewer than 10 studies are included, as the statistical test power is too low to reliably distinguish chance from real asymmetry. Consequently, the inability to statistically evaluate publication bias due to our small dataset (n=9) represents a distinct limitation in validating the overall robustness of our pooled findings.

(2) The methodological quality of the included research is inadequate, which significantly limits the reliability of our conclusions. Risk of bias assessment revealed pervasive deficiencies in reporting randomization, allocation concealment, and blinding implementation among the included studies. The absence of allocation concealment and inadequate blinding pose substantial risks of selection and detection biases. These prevalent methodological flaws inevitably affect the robustness of our pooled results. Consequently, our findings are admittedly impacted by these biases and must be interpreted strictly as preliminary evidence. We strongly emphasize that future experimental studies must strictly adhere to standardized reporting guidelines, ensuring rigorous randomization, proper allocation concealment, and effective blinding to provide high-quality evidence that can robustly verify these preliminary conclusions. Furthermore, the limited number of included studies (9 studies, 386 animals) and small sample sizes in individual studies resulted in inadequate statistical power, with some exploratory subgroups containing only a single study. Crucially, this small dataset completely precluded the use of meta-regression to systematically investigate the sources of the high statistical heterogeneity observed in key outcomes like NE (I^2^ = 89%), DA (I^2^ = 81%), and ACh (I^2^ = 91%). According to the Cochrane Handbook for Systematic Reviews of Interventions, meta-regression requires a minimum of 10 studies per investigated covariate to avoid spurious associations (Higgins et al., 2024). Because our meta-analysis only included 9 studies in total, conducting meta-regression was statistically unfeasible. This constraint severely hindered our ability to disentangle the overlapping effects of biological factors (species, models) and methodological factors (acupuncture parameters), thereby affecting the robustness and precision of our mechanistic conclusions.

(3) Heterogeneity in intervention protocols and animal models. The included studies exhibited significant heterogeneity regarding acupuncture intervention parameters and the selection of animal models. There is a profound lack of standardized protocols for acupuncture modalities (MA and EA), operational parameters (manipulation techniques, needle insertion depth, and electrical stimulation frequency or intensity), and acupoint combinations. Although GV20 served as a core, high-frequency acupoint, inconsistent supplementary acupoint protocols may directly alter the regulatory effects on monoaminergic neurotransmitters, constituting a major source of heterogeneity in NE, DA, and ACh levels. Concurrently, differences in animal species, modeling methodologies, and sex distributions can lead to substantial variations in the location, severity, and pathological patterns of brain injury. Collectively, these variables complicate the integration of evidence and restrict the identification of an optimal acupuncture intervention protocol.

(4) Inconsistent outcome measurement techniques and limited mechanistic exploration. The analytical methods utilized for outcome measures lack unified standards, with varying techniques (e.g., ELISA versus HPLC) employed across the included studies. Inherent differences in the sensitivity and specificity of these assays may introduce substantial data heterogeneity. Additionally, behavioral indicators such as step-down latency and the number of step-down errors exhibited extremely high heterogeneity. While it is hypothesized that this variance relates to the differential sensitivities of the behavioral paradigms, alongside the animals’ emotional states and stress responses, the limited number of studies prevents the rigorous validation of this hypothesis. Regarding mechanistic exploration, although this study consolidated evidence supporting acupuncture’s regulation of synaptic plasticity via monoamine neurotransmitters, the primary literature did not comprehensively investigate the specific actions of monoamine receptor subtypes or their interactions within broader upstream and downstream signaling cascades. Moreover, the precise regulatory targets of acupuncture concerning monoamine synthesis, release, and reuptake remain incompletely elucidated. Although relevant pathways were analyzed by synthesizing broader literature on acupuncture’s neuromodulatory effects, direct mechanistic research within VaD animal models remains scarce, thereby impeding a more profound understanding of the underlying molecular mechanisms.

(5) Limited external and translational validity. All subjects in this review were rodent VaD models. The pathophysiological characteristics of these models, including abnormal cerebral blood perfusion and specific neurotransmitter disruption patterns, inherently differ from the complex clinical reality of human VaD patients. Furthermore, the included animal studies could not account for multifaceted clinical variables such as disease subtypes, varying severity levels, and patient comorbidities. Crucially, the highly standardized acupuncture protocols utilized in animal experimentation differ fundamentally from the individualized, syndrome differentiation-based approaches employed in real-world clinical practice. This discrepancy poses a significant challenge for translating these preclinical findings into clinical applications for the treatment of human VaD. Future research should integrate multiple diverse animal models to more comprehensively validate the multi-target effects of acupuncture and bridge this translational gap.

## 7 Implication

**Figure 6:**
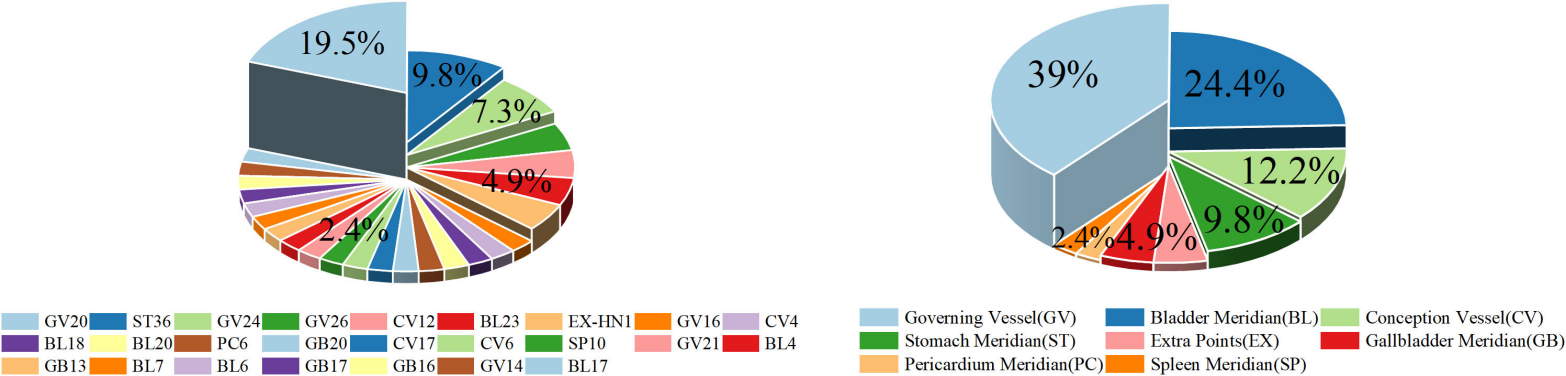
Visualizations of the use of acupuncture points and meridians.

The findings of this study elucidate the regulatory effects of acupuncture on monoaminergic neurotransmitters in VaD animal models, providing a robust reference for subsequent preclinical research and informing future clinical treatment strategies. Initially, an analysis of the acupoint and meridian distribution across the nine included studies (**Figure 6**) revealed that the majority of interventions targeted the Governor Vessel (GV), Bladder Meridian, and Conception Vessel, with GV20, ST36, and GV24 being the most frequently utilized acupoints.

Within the included literature, the frequent selection of GV20, ST36, and GV24 is firmly anchored in both Traditional Chinese Medicine theoretical foundations and modern neurobiological mechanisms, aligning perfectly with the core research theme of monoaminergic regulation. According to Traditional Chinese Medicine principles, the pathological mechanisms of VaD are inextricably linked to the depletion of the ‘sea of marrow’, Qi stagnation accompanied by blood stasis, and phlegm-turbidity obstructing the cerebral orifices (Liu et al., 2024). The GV, conceptualized as the ‘sea of yang meridians’, directly ascends to and communicates with the brain and marrow. As a central acupoint on this meridian, GV20 is traditionally believed to converge yang Qi, replenish essence, and nourish the marrow, thereby restoring cerebral nourishment and improving cognitive function (Yan, 2012; Chen et al., 2016). When applied in conjunction with GV20, GV24 enhances the therapeutic efficacy of tranquilizing the mind, awakening the brain, and unblocking the collaterals. It regulates Qi and blood to stimulate yang Qi, facilitating the replenishment of the ‘sea of marrow’ (Zhou et al., 2019). Furthermore, ST36, functioning as the lower He-sea point of the Stomach Meridian, fortifies the spleen, resolves turbidity, promotes blood circulation, and clears phlegm-turbidity from the brain’s collateral vessels, ultimately ameliorating cognitive impairment (Bi et al., 2016). Anatomically in Traditional Chinese Medicine, the Bladder Meridian runs parallel to the spine and is classically described as entering and connecting with the brain (Yangyang et al., 2022). The Conception Vessel serves as an essential convergence point for Qi and blood, interconnecting with the GV to ensure continuous circulation (Li and Zhang, 2025). The synergistic activation of these specific meridians collectively ameliorates the pathological conditions of Qi-blood deficiency and stagnation characteristic of VaD patients.

From a modern evidence-based perspective, a comprehensive review of VaD indicates that GV20 is the most extensively utilized acupoint in related research, with GV24 and ST36 frequently employed as supplementary points in GV20-based protocols (Feng et al., 2015). Similarly, a previous meta-analysis on post-stroke cognitive impairment demonstrated that acupuncture targeting the GV significantly enhances patients’ cognitive function, further substantiating the therapeutic rationale for prioritizing GV acupuncture in the clinical management of VaD (Han et al., 2024).

Crucially, the present meta-analysis indicates that acupuncture may ameliorate VaD-related cognitive impairment through the systemic regulation of the monoaminergic neurotransmitter network. Our synthesized findings reveal that the targeted stimulation of acupoints such as GV20 and ST36 activates critical monoaminergic nuclei in the central nervous system (including the LC, VTA, and DRN), thereby promoting the synthesis and release of NE, DA, and 5-HT. This neurochemical cascade sequentially modulates corresponding receptors (e.g., D1/D5 receptors for DA, β_1_-AR for NE) and their downstream intracellular signaling pathways (including cAMP/PKA and CREB). These molecular events ultimately facilitate synaptic plasticity in the hippocampus and improve learning and memory behaviors. Notably, with the exception of the study by Xian et al (Xian, 2004), the remaining eight included studies consistently targeted GV20. This high consensus further underscores its pivotal role as a primary neuromodulatory node for monoaminergic regulation in VaD models.

In summary, acupuncture demonstrates a substantial capacity to regulate monoaminergic neurotransmitters in VaD animal models by targeting key acupoints, such as GV20, ST36, and GV24, thereby establishing a critical translational foundation for subsequent clinical research. However, methodological heterogeneity in current preclinical studies, particularly regarding acupuncture parameters (**Supplementary Material 4**) and neurotransmitter detection, can limit experimental reproducibility and precise interpretation. Therefore, we advocate for methodological improvements in future studies across the following four aspects (1): strict adherence to the ARRIVE guidelines for transparent parameter reporting (Percie du Sert et al., 2020) (2); detailed descriptions of specific interventions (e.g., acupuncture type, frequency, and intensity, among other essential parameters) (3); objective quantification of manual acupuncture via standardized single-operator protocols to minimize bias; and (4) establishment of an expert consensus on rodent acupoint localization utilizing advanced visualization techniques. Furthermore, future research should incorporate appropriate sham acupuncture controls and multi-technological approaches for the real-time monitoring of neurotransmitters. Combining these standardization strategies with advanced neurobiological detection and larger sample sizes will significantly enhance experimental credibility and robustly support mechanistic studies on acupuncture for VaD.

## 8 Conclusion

Current preclinical evidence indicates that acupuncture may ameliorate cognitive impairment in VaD by modulating monoaminergic neurotransmitter systems and enhancing synaptic plasticity. However, due to substantial inter-study heterogeneity and small sample sizes, these findings remain exploratory in nature, and the proposed mechanistic pathways should not be overgeneralized. Further methodologically rigorous experimental and clinical studies are urgently warranted to definitively elucidate these mechanisms and substantiate the translational potential of acupuncture in VaD.

## Data Availability

The original contributions presented in the study are included in the article/**Supplementary Material**. Further inquiries can be directed to the corresponding authors.

## Supplementary material

Supplementary Material 1 Search strategy.

Supplementary Material 2 Sensitivity analysis diagram.

Supplementary Material 3 Original Forest plots.

Supplementary Material 4 Structured analysis of heterogeneity.
