# Supplementary material for: Effect of acupuncture on monoaminergic neurotransmitters in animal models of vascular dementia: a preclinical systematic review and meta-analysis
Source: Front Physiol. 2026 May 11;17:1811438. doi: 10.3389/fphys.2026.1811438 (PMC13198999; doi:10.3389/fphys.2026.1811438)
Supplement: Supplementary Material 1 — Search strategy. [file Supplementaryfile1.docx]

**Effect of acupuncture on monoaminergic neurotransmitters in animal models of vascular dementia: a preclinical systematic review and meta-analysis**

**Supplementary File 1** Search strategy.

**Table 1:** PubMed Search

| **NO** | **Search Details** | **Results** |
| --- | --- | --- |
| #1 | ("Acupuncture"[Mesh]) OR ("Acupuncture Therapy"[Mesh]) | 32,996 |
| #2 | ((((((((((Acupuncture[Title/Abstract]) OR (Acupuncture Treatment[Title/Abstract])) OR (Acupuncture Treatments[Title/Abstract])) OR (Treatment, Acupuncture[Title/Abstract])) OR (Therapy, Acupuncture[Title/Abstract])) OR (Pharmacoacupuncture Treatment[Title/Abstract])) OR (Treatment, Pharmacoacupuncture[Title/Abstract])) OR (Pharmacoacupuncture Therapy[Title/Abstract])) OR (Therapy, Pharmacoacupuncture[Title/Abstract])) OR (Electroacupuncture[Title/Abstract])) OR (electro-acupuncture[Title/Abstract]) | 35,677 |
| #3 | #1 OR #2 | 43,249 |
| #4 | ("Dementia, Multi-Infarct"[Mesh]) OR ("Dementia, Vascular"[Mesh]) | 8,073 |
| #5 | ((((((((((((((((((((((((((((((((((((((((((((((Dementias, Vascular[Title/Abstract]) OR (Vascular Dementias[Title/Abstract])) OR (Vascular Dementia[Title/Abstract])) OR (Arteriosclerotic Dementia[Title/Abstract])) OR (Arteriosclerotic Dementias[Title/Abstract])) OR (Dementia, Arteriosclerotic[Title/Abstract])) OR (Dementias, Arteriosclerotic[Title/Abstract])) OR (Binswanger Disease[Title/Abstract])) OR (Disease, Binswanger[Title/Abstract])) OR (Encephalopathy, Binswanger[Title/Abstract])) OR (Chronic Progressive Subcortical Encephalopathy[Title/Abstract])) OR (Binswanger Encephalopathy[Title/Abstract])) OR (Leukoencephalopathy, Subcortical[Title/Abstract])) OR (Leukoencephalopathies, Subcortical[Title/Abstract])) OR (Subcortical Leukoencephalopathies[Title/Abstract])) OR (Encephalopathy, Subcortical Arteriosclerotic[Title/Abstract])) OR (Encephalopathy, Chronic Progressive Subcortical[Title/Abstract])) OR (Encephalopathy, Subcortical, Chronic Progressive[Title/Abstract])) OR (Subcortical Encephalopathy, Chronic Progressive[Title/Abstract])) OR (Subcortical Leukoencephalopathy[Title/Abstract])) OR (Subcortical Arteriosclerotic Encephalopathy[Title/Abstract])) OR (Arteriosclerotic Encephalopathy, Subcortical[Title/Abstract])) OR (Arteriosclerotic Encephalopathies, Subcortical[Title/Abstract])) OR (Encephalopathies, Subcortical Arteriosclerotic[Title/Abstract])) OR (Subcortical Arteriosclerotic Encephalopathies[Title/Abstract])) OR (Encephalopathy, Binswanger's[Title/Abstract])) OR (Binswanger's Encephalopathy[Title/Abstract])) OR (Encephalopathy, Binswangers[Title/Abstract])) OR (Binswanger's Disease[Title/Abstract])) OR (Binswangers Disease[Title/Abstract])) OR (Disease, Binswanger's[Title/Abstract])) OR (Subcortical Vascular Dementia[Title/Abstract])) OR (Dementias, Subcortical Vascular[Title/Abstract])) OR (Dementia, Subcortical Vascular[Title/Abstract])) OR (Subcortical Vascular Dementias[Title/Abstract])) OR (Vascular Dementias, Subcortical[Title/Abstract])) OR (Vascular Dementia, Subcortical[Title/Abstract])) OR (Vascular Dementia, Acute Onset[Title/Abstract])) OR (Acute Onset Vascular Dementia[Title/Abstract])) OR (Dementia, Multi-Infarct[Title/Abstract])) OR (multi-infarct dementia[Title/Abstract])) OR (multiinfarct dementia[Title/Abstract])) OR (infarct dementia[Title/Abstract])) OR (post-stroke dementia[Title/Abstract])) OR (poststroke dementia[Title/Abstract])) OR (stroke dementia[Title/Abstract])) OR (vascular cognitive impairment[Title/Abstract]) | 15,206 |
| #6 | #4 OR #5 | 17,220 |
| #7 | ("Models, Animal"[Mesh]) OR "Animal Experimentation"[Mesh] | 700,832 |
| #8 | "rat"[Title/Abstract] OR "rats"[Title/Abstract] OR "mouse"[Title/Abstract] OR "mice"[Title/Abstract] OR "rabbit"[Title/Abstract] OR "rabbits"[Title/Abstract] OR "dog"[Title/Abstract] OR "pig"[Title/Abstract] OR "animal"[Title/Abstract] OR "animals"[Title/Abstract] OR "experiment*"[Title/Abstract] | 6,339,678 |
| #9 | #7 OR #8 | 6,455,611 |
| #10 | #3 AND #6 AND #9 | 107 |

**Table 2:** Embase Search

| **NO** | **Search Details** | **Results** |
| --- | --- | --- |
| #1 | 'acupuncture'/exp OR 'acupuncture' | 82,374 |
| #2 | 'acupuncture':ab,ti OR 'acupuncture treatment':ab,ti OR 'acupuncture treatments':ab,ti OR 'treatment, acupuncture':ab,ti OR 'therapy, acupuncture':ab,ti OR 'pharmacoacupuncture treatment':ab,ti OR 'treatment, pharmacoacupuncture':ab,ti OR 'pharmacoacupuncture therapy':ab,ti OR 'therapy, pharmacoacupuncture':ab,ti OR 'electroacupuncture':ab,ti OR 'electro-acupuncture':ab,ti | 50,635 |
| #3 | #1 OR #2 | 82,463 |
| #4 | 'multiinfarct dementia'/exp OR 'multiinfarct dementia' | 16,670 |
| #5 | 'dementias, vascular':ab,ti OR 'vascular dementias':ab,ti OR 'vascular dementia':ab,ti OR 'arteriosclerotic dementia':ab,ti OR 'arteriosclerotic dementias':ab,ti OR 'dementia, arteriosclerotic':ab,ti OR 'dementias, arteriosclerotic':ab,ti OR 'chronic progressive subcortical encephalopathy':ab,ti OR 'leukoencephalopathy, subcortical':ab,ti OR 'leukoencephalopathies, subcortical':ab,ti OR 'subcortical leukoencephalopathies':ab,ti OR 'encephalopathy, subcortical arteriosclerotic':ab,ti OR 'encephalopathy, chronic progressive subcortical':ab,ti OR 'encephalopathy, subcortical, chronic progressive':ab,ti OR 'subcortical encephalopathy, chronic progressive':ab,ti OR 'subcortical leukoencephalopathy':ab,ti OR 'subcortical arteriosclerotic encephalopathy':ab,ti OR 'arteriosclerotic encephalopathy, subcortical':ab,ti OR 'arteriosclerotic encephalopathies, subcortical':ab,ti OR 'encephalopathies, subcortical arteriosclerotic':ab,ti OR 'subcortical arteriosclerotic encephalopathies':ab,ti OR 'encephalopathy, binswanger':ab,ti OR 'binswanger encephalopathy':ab,ti OR 'encephalopathy, binswangers':ab,ti OR 'binswanger disease':ab,ti OR 'binswangers disease':ab,ti OR 'disease, binswanger':ab,ti OR 'subcortical vascular dementia':ab,ti OR 'dementias, subcortical vascular':ab,ti OR 'dementia, subcortical vascular':ab,ti OR 'subcortical vascular dementias':ab,ti OR 'vascular dementias, subcortical':ab,ti OR 'vascular dementia, subcortical':ab,ti OR 'vascular dementia, acute onset':ab,ti OR 'acute onset vascular dementia':ab,ti OR 'dementia, multi-infarct':ab,ti OR 'multi-infarct dementia':ab,ti OR 'multiinfarct dementia':ab,ti OR 'infarct dementia':ab,ti OR 'post-stroke dementia':ab,ti OR 'poststroke dementia':ab,ti OR 'stroke dementia':ab,ti OR ' vascular cognitive impairment':ab,ti | 17,762 |
| #6 | #4 OR #5 | 23,348 |
| #7 | 'animal model'/exp OR 'animal model' | 2,067,719 |
| #8 | 'animal experiment'/exp OR 'animal experiment' | 3,425,777 |
| #9 | 'rat':ti,ab,kw OR 'rats':ti,ab,kw OR 'mouse':ti,ab,kw OR 'mice':ti,ab,kw OR 'rabbit':ti,ab,kw OR 'rabbits':ti,ab,kw OR 'dog':ti,ab,kw OR 'pig':ti,ab,kw OR 'animal':ti,ab,kw OR 'animals':ti,ab,kw OR 'experiment*':ti,ab,kw | 7,497,297 |
| #10 | #7 OR #8 OR #9 | 8,363,374 |
| #11 | #3 AND #6 AND #10 | 162 |

**Table 3:** Web of Science Search

| **NO** | **Search Details** | **Results** |
| --- | --- | --- |
| #1 | TS=(‘Acupuncture Therapy’ OR ‘Acupuncture’ OR ‘Acupuncture Treatment’ OR ‘Acupuncture Treatments’ OR ‘Treatment, Acupuncture’ OR ‘Therapy, Acupuncture’ OR ‘Pharmacoacupuncture Treatment’ OR ‘Treatment, Pharmacoacupuncture’ OR ‘Pharmacoacupuncture Therapy’ OR ‘Therapy, Pharmacoacupuncture’ OR ‘Electroacupuncture’ OR ‘electro-acupuncture’) | 64,872 |
| #2 | TS=(‘Dementia, Vascular’ OR ‘Dementias, Vascular’ OR ‘Vascular Dementias’ OR ‘Vascular Dementia’ OR ‘Arteriosclerotic Dementia’ OR ‘Arteriosclerotic Dementias’ OR ‘Dementia, Arteriosclerotic’ OR ‘Dementias, Arteriosclerotic’ OR ‘Binswanger Disease’ OR ‘Disease, Binswanger’ OR ‘Encephalopathy, Binswanger’ OR ‘Chronic Progressive Subcortical Encephalopathy’ OR ‘Binswanger Encephalopathy’ OR ‘Leukoencephalopathy, Subcortical’ OR ‘Leukoencephalopathies, Subcortical’ OR ‘Subcortical Leukoencephalopathies’ OR ‘Encephalopathy, Subcortical Arteriosclerotic’ OR ‘Encephalopathy, Chronic Progressive Subcortical’ OR ‘Encephalopathy, Subcortical, Chronic Progressive’ OR ‘Subcortical Encephalopathy, Chronic Progressive’ OR ‘Subcortical Leukoencephalopathy’ OR ‘Subcortical Arteriosclerotic Encephalopathy’ OR ‘Arteriosclerotic Encephalopathy, Subcortical’ OR ‘Arteriosclerotic Encephalopathies, Subcortical’ OR ‘Encephalopathies, Subcortical Arteriosclerotic’ OR ‘Subcortical Arteriosclerotic Encephalopathies’ OR ‘Encephalopathy, Binswanger's’ OR ‘Binswanger's Encephalopathy’ OR ‘Encephalopathy, Binswangers’ OR ‘Binswanger's Disease’ OR ‘Binswangers Disease’ OR ‘Disease, Binswanger's’ OR ‘Subcortical Vascular Dementia’ OR ‘Dementias, Subcortical Vascular’ OR ‘Dementia, Subcortical Vascular’ OR ‘Subcortical Vascular Dementias’ OR ‘Vascular Dementias, Subcortical’ OR ‘Vascular Dementia, Subcortical’ OR ‘Vascular Dementia, Acute Onset’ OR ‘Acute Onset Vascular Dementia’ OR ‘Dementia, Multi-Infarct’ OR ‘multi-infarct dementia’ OR ‘multiinfarct dementia’ OR ‘infarct dementia’ OR ‘post-stroke dementia’ OR ‘poststroke dementia’ OR ‘stroke dementia’ OR ‘vascular cognitive impairment’) | 71,708 |
| #3 | TS=(‘animal model’ OR ‘animal experimentation’ OR ‘rat’ OR ‘rats’ OR ‘mouse’ OR ‘mice’ OR ‘rabbit’ OR ‘rabbits’ OR ‘dog’ OR ‘pig’ OR ‘animal*’ OR ‘experiment*’) | 31,010,755 |
| #4 | #1 AND #2 AND #3 | 277 |

**Table 4:** Cochrane Library Search

| NO | Search Details | Results |
| --- | --- | --- |
| #1 | MeSH descriptor: [Acupuncture Therapy] explode all trees | 7,244 |
| #2 | MeSH descriptor: [Acupuncture] explode all trees | 216 |
| #3 | “Acupuncture":ti,ab,kw OR "Acupuncture Treatment”:ti,ab,kw OR “Acupuncture Treatments”:ti,ab,kw OR “Treatment, Acupuncture”:ti,ab,kw OR “Therapy, Acupuncture”:ti,ab,kw OR “Pharmacoacupuncture Treatment”:ti,ab,kw OR “Treatment, Pharmacoacupuncture”:ti,ab,kw OR “Pharmacoacupuncture Therapy”:ti,ab,kw OR “Therapy, Pharmacoacupuncture”:ti,ab,kw OR “Electroacupuncture”:ti,ab,kw OR “electro-acupuncture”:ti,ab,kw | 23,316 |
| #4 | MeSH descriptor: [Dementia, Vascular] explode all trees | 461 |
| #5 | MeSH descriptor: [Dementia, Multi-Infarct] explode all trees | 77 |
| #6 | (“Dementias, Vascular” OR “Vascular Dementias” OR “Vascular Dementia” OR “Arteriosclerotic Dementia” OR “Arteriosclerotic Dementias” OR “Dementia, Arteriosclerotic” OR “Dementias, Arteriosclerotic” OR “Binswanger Disease” OR “Disease, Binswanger” OR “Encephalopathy, Binswanger” OR “Chronic Progressive Subcortical Encephalopathy” OR “Binswanger Encephalopathy” OR “Leukoencephalopathy, Subcortical” OR “Leukoencephalopathies, Subcortical” OR “Subcortical Leukoencephalopathies” OR “Encephalopathy, Subcortical Arteriosclerotic” OR “Encephalopathy, Chronic Progressive Subcortical” OR “Encephalopathy, Subcortical, Chronic Progressive” OR “Subcortical Encephalopathy, Chronic Progressive” OR “Subcortical Leukoencephalopathy” OR “Subcortical Arteriosclerotic Encephalopathy” OR “Arteriosclerotic Encephalopathy, Subcortical” OR “Arteriosclerotic Encephalopathies, Subcortical” OR “Encephalopathies, Subcortical Arteriosclerotic” OR “Subcortical Arteriosclerotic Encephalopathies” OR “Encephalopathy, Binswanger's” OR “Binswanger's Encephalopathy” OR “Encephalopathy, Binswangers” OR “Binswanger's Disease” OR “Binswangers Disease” OR “Disease, Binswanger's” OR “Subcortical Vascular Dementia” OR “Dementias, Subcortical Vascular” OR “Dementia, Subcortical Vascular” OR “Subcortical Vascular Dementias” OR “Vascular Dementias, Subcortical” OR “Vascular Dementia, Subcortical” OR “Vascular Dementia, Acute Onset” OR “Acute Onset Vascular Dementia” OR “Dementia, Multi-Infarct” OR “multi-infarct dementia” OR “multiinfarct dementia” OR “infarct dementia” OR “post-stroke dementia” OR “poststroke dementia” OR “stroke dementia” OR “vascular cognitive impairment” ):ti,ab,kw | 1,477 |
| #7 | MeSH descriptor: [Models, Animal] explode all trees | 1,044 |
| #8 | MeSH descriptor: [Animal Experimentation] explode all trees | 10 |
| #9 | rat:ti,ab,kw OR rats:ti,ab,kw OR mouse:ti,ab,kw OR mice:ti,ab,kw OR rabbit:ti,ab,kw OR rabbits:ti,ab,kw OR dog:ti,ab,kw OR pig:ti,ab,kw OR animal:ti,ab,kw OR animals:ti,ab,kw OR experiment*:ti,ab,kw | 223,837 |
| #10 | #1 OR #2 OR #3 | 23,512 |
| #11 | #4 OR #5 OR #6 | 1,609 |
| #12 | #7 OR #8 OR #9 | 223,837 |
| #13 | #10 AND #11 AND #12 | 16 |

**Table 5:** CNKI Search

| **NO** | **Search Details** | **Results** |
| --- | --- | --- |
| #1 | (TKA=(‘针刺’+‘针灸’+‘手针’+‘电针’) OR SU=(‘针刺疗法’+‘针刺’+‘针灸疗法’)) AND (TKA=(‘血管性痴呆’+‘动脉硬化性痴呆’+‘梗塞性痴呆’+‘梗死性痴呆’+‘卒中后痴呆’+‘宾斯旺格病’+‘血管性认知障碍’+‘血管性认知损害’+‘血管性认知损伤’) OR SU=(‘痴呆, 血管性’+‘痴呆, 多发性梗死性’)) AND (TKA=(‘动物’+‘鼠’+‘兔’+‘狗’+‘猪’+ ‘基础研究’) OR SU=(‘动物模型’+ ‘动物实验’)) | 485 |

**Table 6:** Wanfang Search

| **NO** | **Search Details** | **Results** |
| --- | --- | --- |
| #1 | (题名或关键词:(针刺 OR 针灸OR 手针 OR 电针) OR 主题:(针刺疗法 OR 针灸疗法 OR 针刺)) and (题名或关键词:(血管性痴呆 OR 动脉硬化性痴呆 OR 梗塞性痴呆OR 梗死性痴呆 OR卒中后痴呆OR 宾斯旺格病OR血管性认知障碍 OR 血管性认知损害OR 血管性认知损伤) OR 主题:(痴呆，血管性 OR 痴呆, 多发性梗死性)) and (题名或关键词:(动物 OR 鼠 OR 兔 OR 狗 OR 猪OR 基础研究) OR 主题:(动物实验 OR 动物模型)) | 414 |

**Table 7:** cqvip Search

| **NO** | **Search Details** | **Results** |
| --- | --- | --- |
| #1 | M=(针刺 OR 针灸OR 手针 OR 电针 OR 针刺疗法 OR 针灸疗法) and M=(血管性痴呆 OR 动脉硬化性痴呆 OR 梗塞性痴呆OR 梗死性痴呆 OR卒中后痴呆 OR 宾斯旺格病 OR血管性认知障碍 OR 血管性认知损害OR 血管性认知损伤) and R=(动物 OR 鼠 OR 兔 OR 狗 OR 猪 OR 基础研究) | 262 |

**Table 8:** CBM Search

| **NO** | **Search Details** | **Results** |
| --- | --- | --- |
| #1 | "针刺疗法"[加权:扩展] OR "针刺"[加权:扩展] OR "针灸疗法"[加权:扩展] | 157,820 |
| #2 | "针刺"[常用字段:智能] OR "针灸"[常用字段:智能] OR "手针"[常用字段:智能] OR "电针"[常用字段:智能] | 285,580 |
| #3 | (#2) OR (#1) | 287,352 |
| #4 | "痴呆, 血管性" [加权:扩展] OR "痴呆, 多发性梗死性"[加权:扩展] | 10,946 |
| #5 | "血管性痴呆"[常用字段:智能] OR "动脉硬化性痴呆"[常用字段:智能] OR "梗塞性痴呆"[常用字段:智能] OR "梗死性痴呆"[常用字段:智能] OR "卒中后痴呆"[常用字段:智能] OR "宾斯旺格病"[常用字段:智能] OR "血管性认知障碍" OR "血管性认知损害"[常用字段:智能] OR "血管性认知损伤"[常用字段:智能] | 15,125 |
| #6 | (#5) OR (#4) | 15,125 |
| #7 | "动物模型" [加权:扩展] OR "动物实验"[加权:扩展] | 43,337 |
| #8 | "动物"[常用字段:智能] OR "鼠"[常用字段:智能] OR "兔"[常用字段:智能] OR "狗"[常用字段:智能] OR "猪"[常用字段:智能] OR "基础研究"[常用字段:智能] | 1,028,600 |
| #9 | (#8) OR (#7) | 1,029,866 |
| #10 | (#9) AND (#6) AND (#3) | 396 |
