# Supplementary material for: Effect of acupuncture on monoaminergic neurotransmitters in animal models of vascular dementia: a preclinical systematic review and meta-analysis
Source: Front Physiol. 2026 May 11;17:1811438. doi: 10.3389/fphys.2026.1811438 (PMC13198999; doi:10.3389/fphys.2026.1811438)

**Effect of acupuncture on monoaminergic neurotransmitters in animal models of vascular dementia: a preclinical systematic review and meta-analysis**

**Supplementary Material 2** Sensitivity analysis diagram.

1. NE


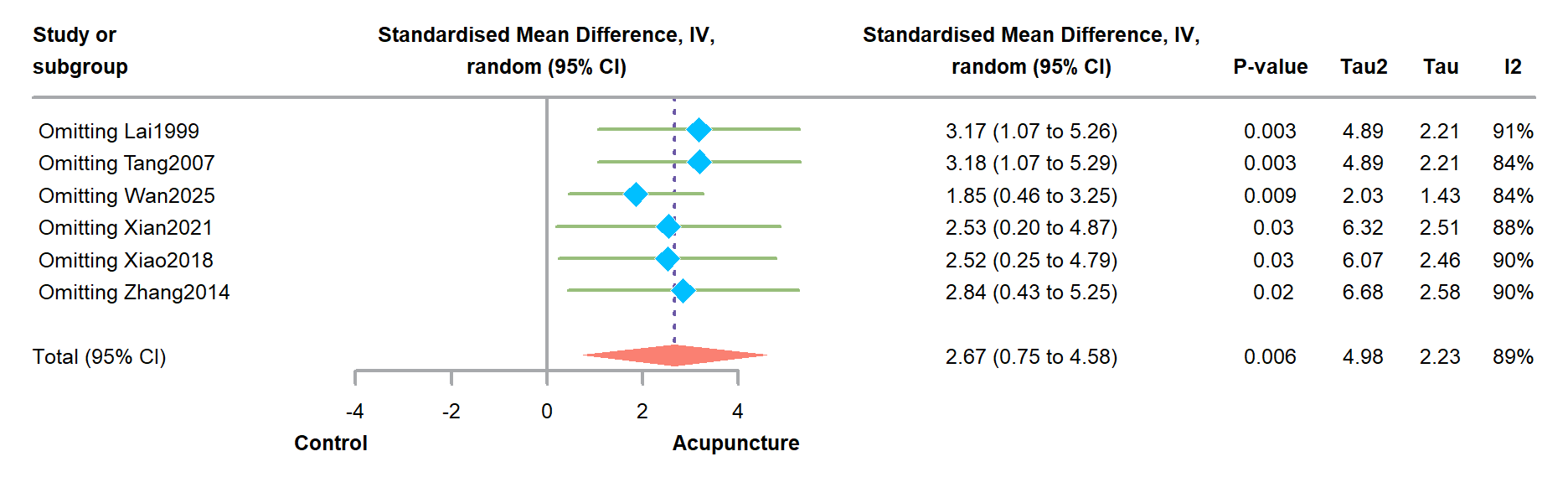


2. DA


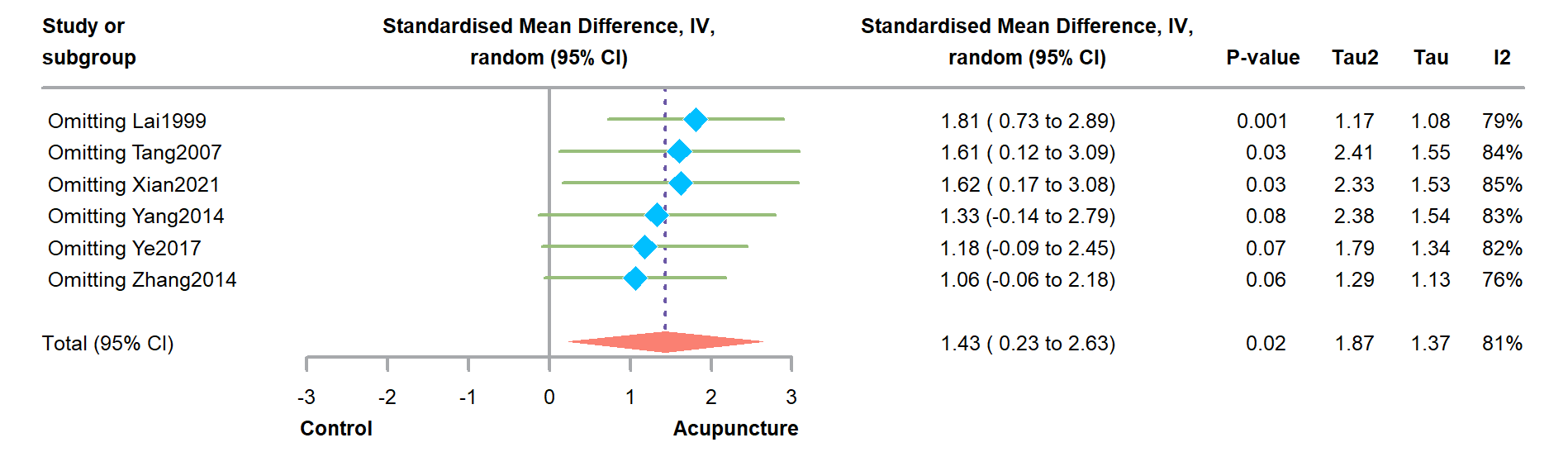


3. ACh


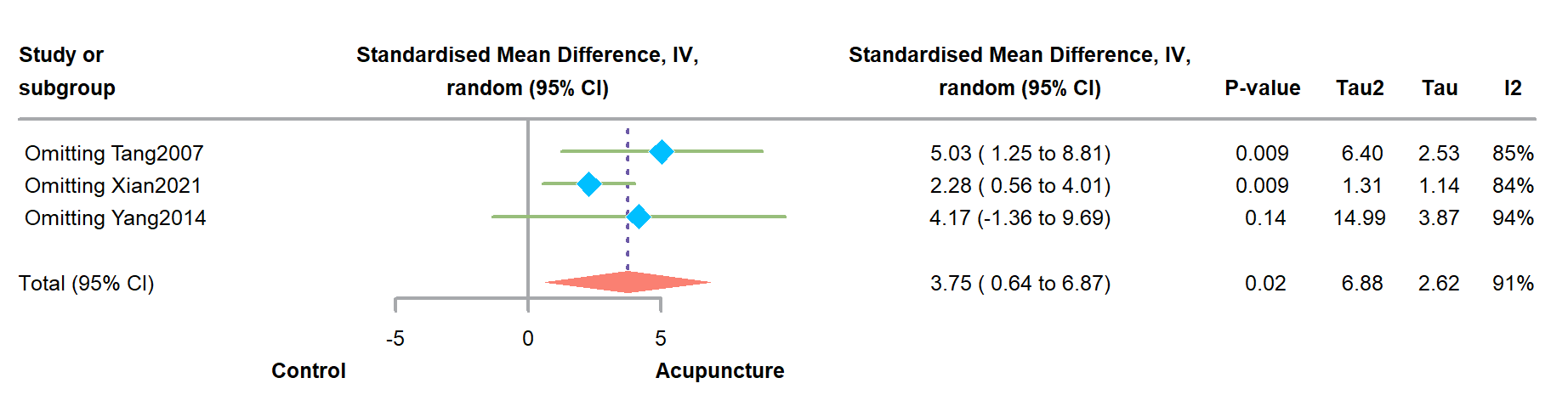


4. MWM-Escape latency


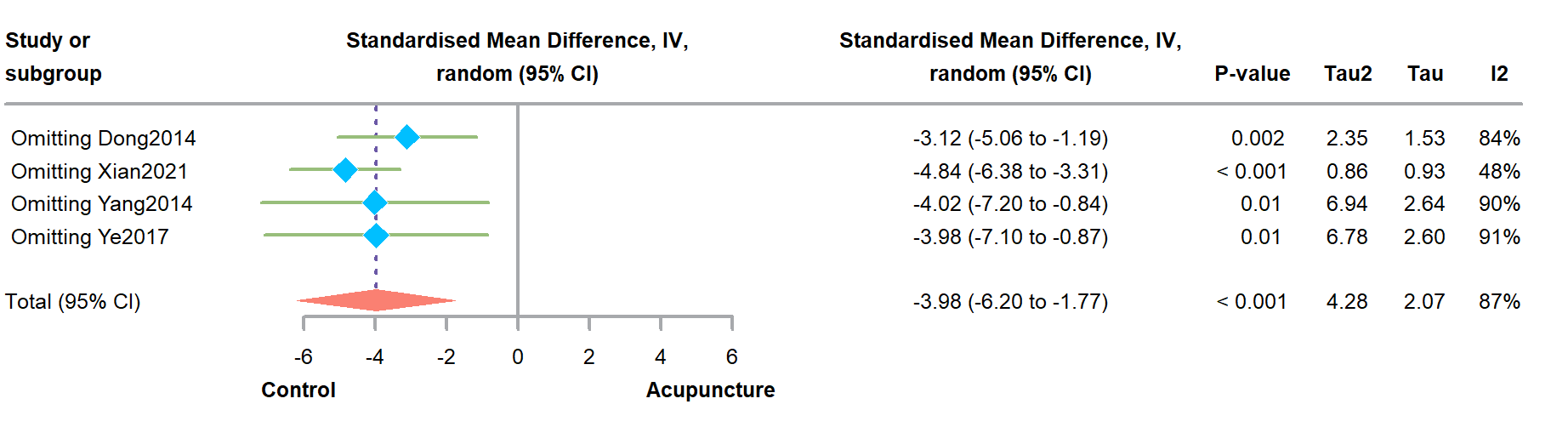

Supplement: Supplementary Material 2 — Sensitivity analysis diagram. [file Supplementaryfile2.docx]
