# Supplementary material for: Effect of acupuncture on monoaminergic neurotransmitters in animal models of vascular dementia: a preclinical systematic review and meta-analysis
Source: Front Physiol. 2026 May 11;17:1811438. doi: 10.3389/fphys.2026.1811438 (PMC13198999; doi:10.3389/fphys.2026.1811438)

**Effect of acupuncture on monoaminergic neurotransmitters in animal models of vascular dementia: a preclinical systematic review and meta-analysis**

**Supplementary Material 3** Original forest plots.

1. 5-HT


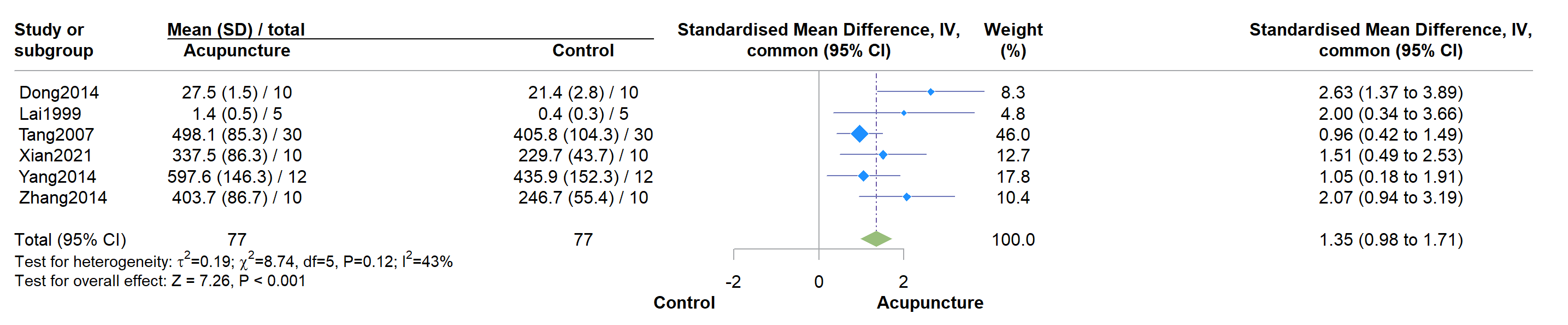


2. NE


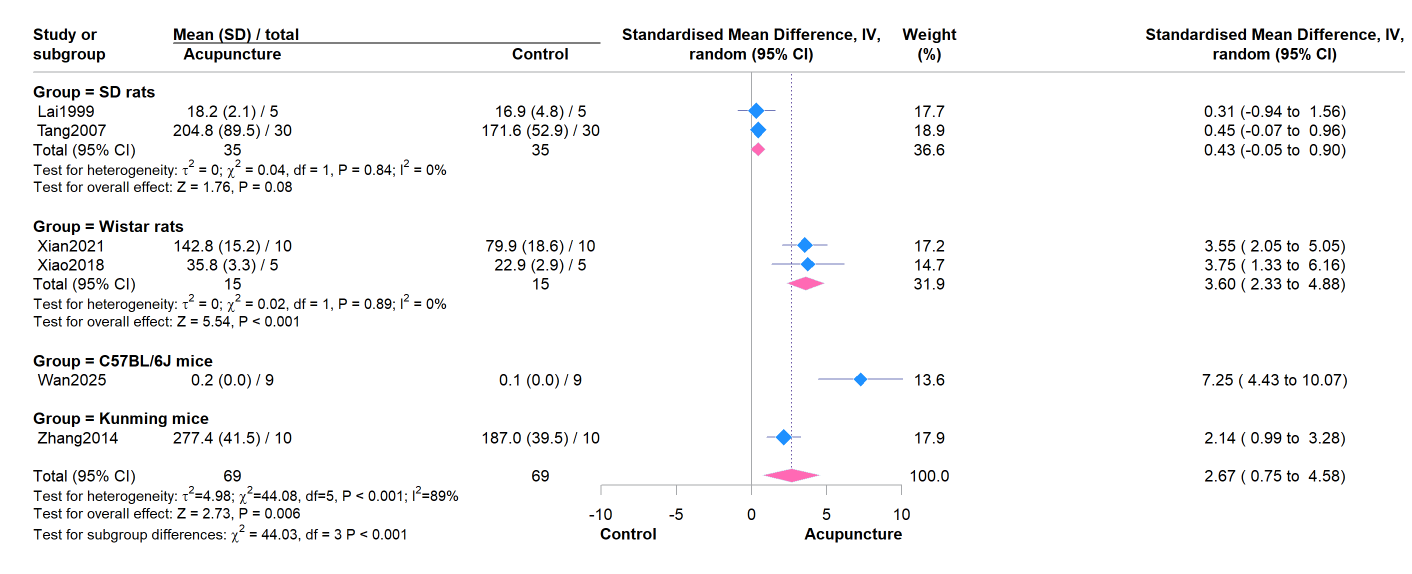


3. DA


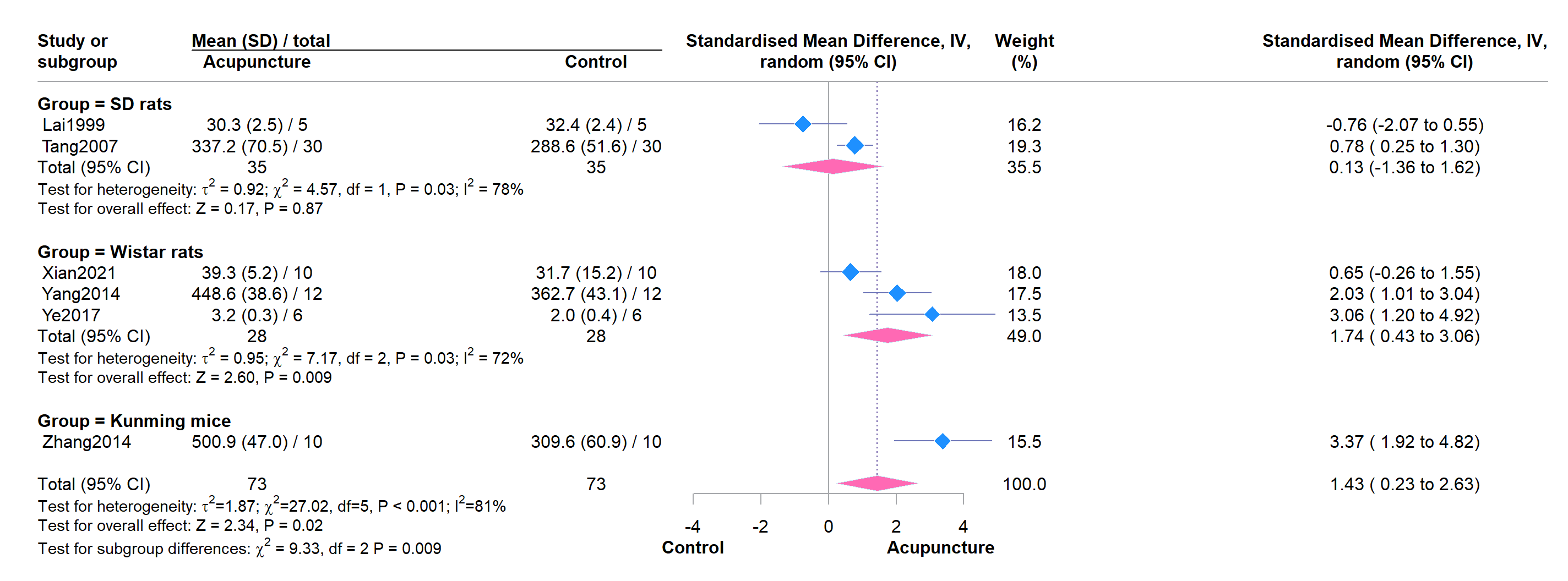


4. ACh


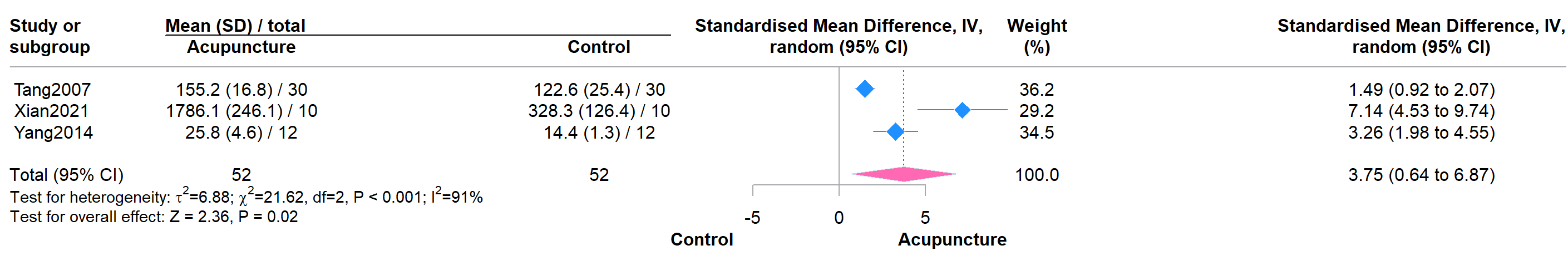


5. LTP


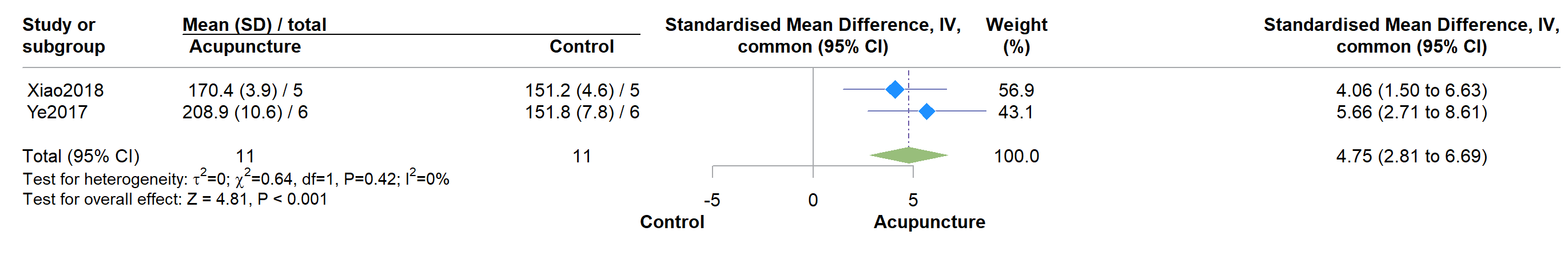


6. MWM-Escape latency


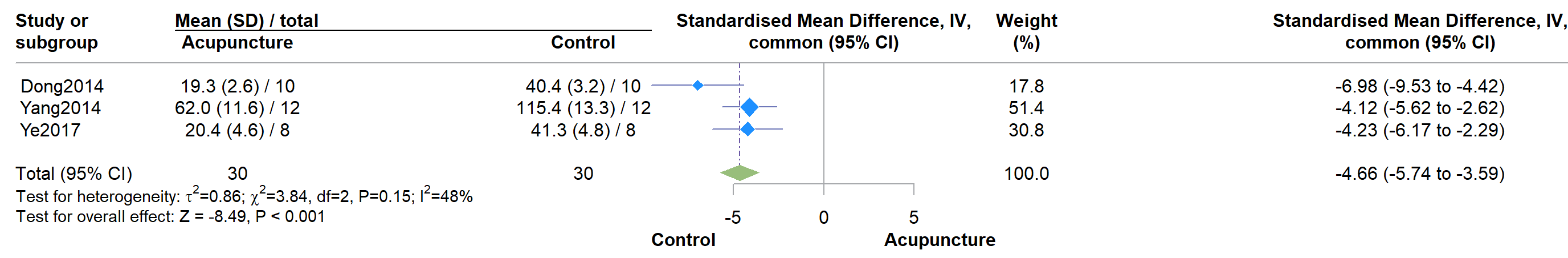


7. MWM-Number of platform crossings


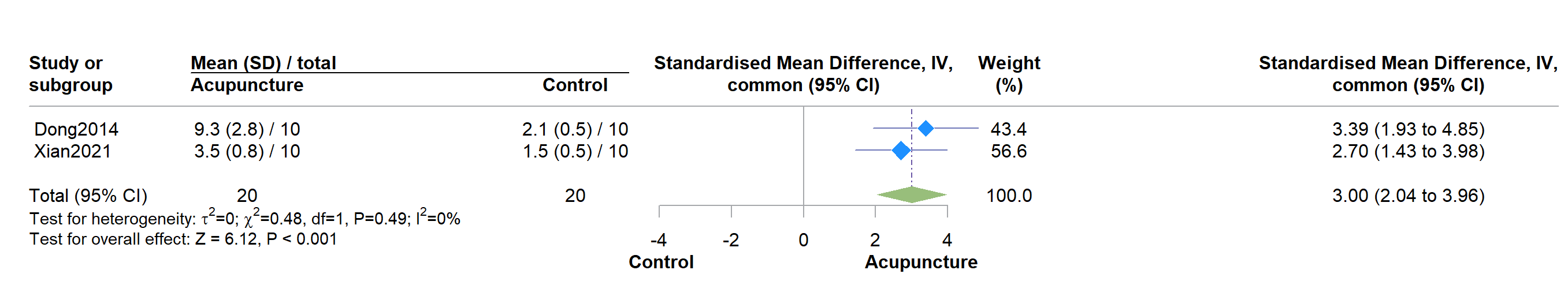


8. Step-Down Latency


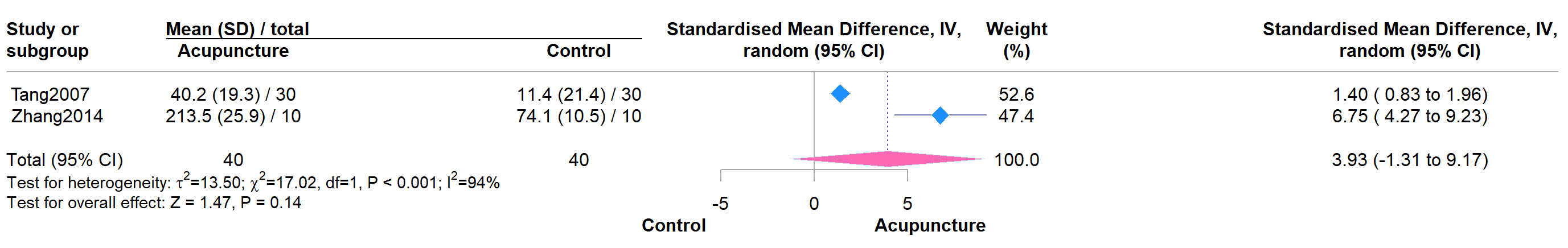


9. Number of Step-Down Errors


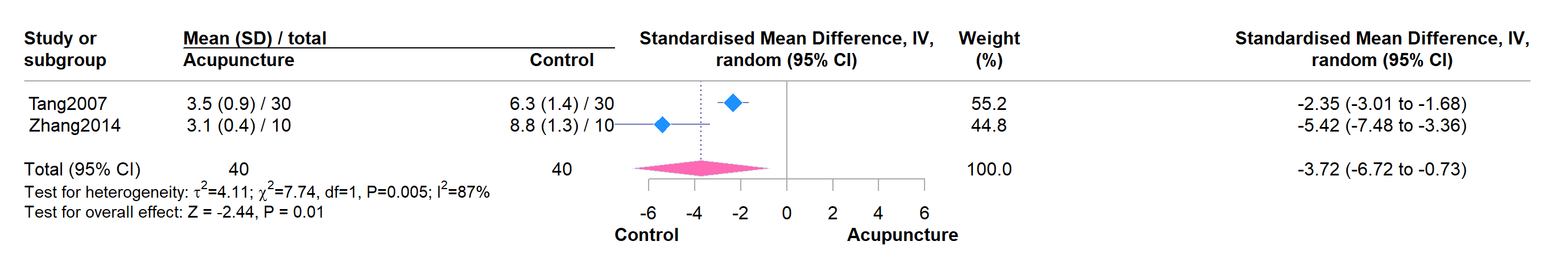

Supplement: Supplementary Material 3 — Original Forest plots. [file Supplementaryfile3.docx]
