# Supplementary material for: Effect of acupuncture on monoaminergic neurotransmitters in animal models of vascular dementia: a preclinical systematic review and meta-analysis
Source: Front Physiol. 2026 May 11;17:1811438. doi: 10.3389/fphys.2026.1811438 (PMC13198999; doi:10.3389/fphys.2026.1811438)
Supplement: Supplementary Material 4 — Structured analysis of heterogeneity. [file Supplementaryfile4.docx]

**Effect of acupuncture on monoaminergic neurotransmitters in animal models of vascular dementia: a preclinical systematic review and meta-analysis**

**Supplementary Material 4** Structured analysis of heterogeneity.

| Study | Biological Heterogeneity (Animal Species, Sex, Age/Weight) | Biological Heterogeneity (VaD Modeling Method) | Methodological Heterogeneity (Acupuncture Modality & Acupoints) | Methodological Heterogeneity (Core Parameters:Frequency/Intensity/  Duration/Manipulation) |
| --- | --- | --- | --- | --- |
| Dong et al. (2014) | Sprague Dawley rats, Male, 2–3 months, 200 ± 20g | Modified 2VO combined with SNP | MA  (GV16, GV20, GV26, CV12, CV16) | 20 min, once daily for 15 days; Depth ~25 mm, even reinforcement and reduction method |
| Lai et al. (1999) | Sprague Dawley rats, Male, 200-250g | 2VO | EA  (GV20, BL23) | 10 min, once every 12h starting 24h post-modeling for 3 sessions; Dense-Sparse Wave, 10-12 Hz, 6-10V |
| Tang et al. (2007) | Sprague Dawley rats, Male, 4–5 months, 250-300g | Modified 2VO combined with SNP | MA  (BL23, BL18, BL20, GV20, EX-HN1, GV24, GV26, PC6, GB20) | 30 min, once daily for 3 courses (10 days/course) with 1-day intervals; Twisting and lifting interpolation |
| Wan et al. (2025) | C57BL/6J mice, Male, 6–8 weeks | BCAS | EA  (GV20, GV24) | 20 min, once every other day for 4 weeks; Alternating frequency 2/15 Hz, 2 mA |
| Xian (2021) | Wistar rats, Male/Female, 10 months, 300 ± 40g | Thromboembolic Multiple Infarction Dementia Model | MA  (CV17, CV12, CV6, SP10, ST36) | Once daily for 7 days; 30s reinforcing method (reducing for SP10) |
| Xiao et al. (2018) | Wistar rats, Male, 7–8 weeks, 270-300g | 2VO | MA  (GV20, ST36) | 10 min, once daily for 2 weeks (1 day rest after 6 treatments); Depth 5 mm |
| Yang et al. (2014) | Wistar rats, Male/Female, 200-250g | 2VO | MA  (GV24, GV20, GV21, EX-HN1, BL4, GB13, BL7, BL6, GB17, GB16) | 6 h, once daily for 4 weeks; Twirling at 200 rpm for 3 min, once hourly |
| Ye et al. (2017) | Wistar rats, Male, 270-300g | 2VO | MA  (GV20, ST36) | Once daily for 2 weeks (1 day rest after 6 treatments); Twirling at 120 rpm for 30 s |
| Zhang (2014) | Kunming mice, Male, 28-35g | 2VO | EA  (GV14, GV20, BL17, ST36) | 10 min, once daily for 15 days; Dense-Sparse Wave, 2-80 Hz |

2VO: Two-Vessel Occlusion; SNP: Sodium nitroprusside; BCAS: Bilateral common carotid artery stenosis; EA: Electroacupuncture; MA: Manual Acupuncture.
